# CARM1 hypermethylates the NuRD chromatin remodeling complex to promote cell cycle gene expression and breast cancer development

**DOI:** 10.1093/nar/gkae329

**Published:** 2024-04-27

**Authors:** Xue Chen, Ming-feng Huang, Da-meng Fan, Yao-hui He, Wen-juan Zhang, Jian-cheng Ding, Bing-ling Peng, Xu Pan, Ya Liu, Jun Du, Ying Li, Zhi-ying Liu, Bing-lan Xie, Zhi-jian Kuang, Jia Yi, Wen Liu

**Affiliations:** State Key Laboratory of Cellular Stress Biology, School of Pharmaceutical Sciences, Faculty of Medicine and Life Sciences, Xiamen University, Xiang’an South Road, Xiamen, Fujian 361102, China; Fujian Provincial Key Laboratory of Innovative Drug Target Research, School of Pharmaceutical Sciences, Faculty of Medicine and Life Sciences, Xiamen University, Xiang’an South Road, Xiamen, Fujian 361102, China; Xiang An Biomedicine Laboratory, School of Pharmaceutical Sciences, Faculty of Medicine and Life Sciences, Xiamen University, Xiang’an South Road, Xiamen, Fujian 361102, China; State Key Laboratory of Cellular Stress Biology, School of Pharmaceutical Sciences, Faculty of Medicine and Life Sciences, Xiamen University, Xiang’an South Road, Xiamen, Fujian 361102, China; Fujian Provincial Key Laboratory of Innovative Drug Target Research, School of Pharmaceutical Sciences, Faculty of Medicine and Life Sciences, Xiamen University, Xiang’an South Road, Xiamen, Fujian 361102, China; State Key Laboratory of Cellular Stress Biology, School of Pharmaceutical Sciences, Faculty of Medicine and Life Sciences, Xiamen University, Xiang’an South Road, Xiamen, Fujian 361102, China; Fujian Provincial Key Laboratory of Innovative Drug Target Research, School of Pharmaceutical Sciences, Faculty of Medicine and Life Sciences, Xiamen University, Xiang’an South Road, Xiamen, Fujian 361102, China; Xiang An Biomedicine Laboratory, School of Pharmaceutical Sciences, Faculty of Medicine and Life Sciences, Xiamen University, Xiang’an South Road, Xiamen, Fujian 361102, China; State Key Laboratory of Cellular Stress Biology, School of Pharmaceutical Sciences, Faculty of Medicine and Life Sciences, Xiamen University, Xiang’an South Road, Xiamen, Fujian 361102, China; Fujian Provincial Key Laboratory of Innovative Drug Target Research, School of Pharmaceutical Sciences, Faculty of Medicine and Life Sciences, Xiamen University, Xiang’an South Road, Xiamen, Fujian 361102, China; State Key Laboratory of Cellular Stress Biology, School of Pharmaceutical Sciences, Faculty of Medicine and Life Sciences, Xiamen University, Xiang’an South Road, Xiamen, Fujian 361102, China; Fujian Provincial Key Laboratory of Innovative Drug Target Research, School of Pharmaceutical Sciences, Faculty of Medicine and Life Sciences, Xiamen University, Xiang’an South Road, Xiamen, Fujian 361102, China; Department of Laboratory Medicine, First Affiliated Hospital of Gannan Medical University, No. 23, Qingnian Road, Ganzhou, Jiangxi 341000, China; State Key Laboratory of Cellular Stress Biology, School of Pharmaceutical Sciences, Faculty of Medicine and Life Sciences, Xiamen University, Xiang’an South Road, Xiamen, Fujian 361102, China; Fujian Provincial Key Laboratory of Innovative Drug Target Research, School of Pharmaceutical Sciences, Faculty of Medicine and Life Sciences, Xiamen University, Xiang’an South Road, Xiamen, Fujian 361102, China; State Key Laboratory of Cellular Stress Biology, School of Pharmaceutical Sciences, Faculty of Medicine and Life Sciences, Xiamen University, Xiang’an South Road, Xiamen, Fujian 361102, China; Fujian Provincial Key Laboratory of Innovative Drug Target Research, School of Pharmaceutical Sciences, Faculty of Medicine and Life Sciences, Xiamen University, Xiang’an South Road, Xiamen, Fujian 361102, China; Xiamen University-Amogene Joint R&D Center for Genetic Diagnostics, School of Pharmaceutical Sciences, Xiang’an South Road, Xiamen, Fujian 361102, China; State Key Laboratory of Cellular Stress Biology, School of Pharmaceutical Sciences, Faculty of Medicine and Life Sciences, Xiamen University, Xiang’an South Road, Xiamen, Fujian 361102, China; Fujian Provincial Key Laboratory of Innovative Drug Target Research, School of Pharmaceutical Sciences, Faculty of Medicine and Life Sciences, Xiamen University, Xiang’an South Road, Xiamen, Fujian 361102, China; State Key Laboratory of Cellular Stress Biology, School of Pharmaceutical Sciences, Faculty of Medicine and Life Sciences, Xiamen University, Xiang’an South Road, Xiamen, Fujian 361102, China; Fujian Provincial Key Laboratory of Innovative Drug Target Research, School of Pharmaceutical Sciences, Faculty of Medicine and Life Sciences, Xiamen University, Xiang’an South Road, Xiamen, Fujian 361102, China; State Key Laboratory of Cellular Stress Biology, School of Pharmaceutical Sciences, Faculty of Medicine and Life Sciences, Xiamen University, Xiang’an South Road, Xiamen, Fujian 361102, China; Fujian Provincial Key Laboratory of Innovative Drug Target Research, School of Pharmaceutical Sciences, Faculty of Medicine and Life Sciences, Xiamen University, Xiang’an South Road, Xiamen, Fujian 361102, China; State Key Laboratory of Cellular Stress Biology, School of Pharmaceutical Sciences, Faculty of Medicine and Life Sciences, Xiamen University, Xiang’an South Road, Xiamen, Fujian 361102, China; Fujian Provincial Key Laboratory of Innovative Drug Target Research, School of Pharmaceutical Sciences, Faculty of Medicine and Life Sciences, Xiamen University, Xiang’an South Road, Xiamen, Fujian 361102, China; State Key Laboratory of Cellular Stress Biology, School of Pharmaceutical Sciences, Faculty of Medicine and Life Sciences, Xiamen University, Xiang’an South Road, Xiamen, Fujian 361102, China; Fujian Provincial Key Laboratory of Innovative Drug Target Research, School of Pharmaceutical Sciences, Faculty of Medicine and Life Sciences, Xiamen University, Xiang’an South Road, Xiamen, Fujian 361102, China; State Key Laboratory of Cellular Stress Biology, School of Pharmaceutical Sciences, Faculty of Medicine and Life Sciences, Xiamen University, Xiang’an South Road, Xiamen, Fujian 361102, China; Fujian Provincial Key Laboratory of Innovative Drug Target Research, School of Pharmaceutical Sciences, Faculty of Medicine and Life Sciences, Xiamen University, Xiang’an South Road, Xiamen, Fujian 361102, China; State Key Laboratory of Cellular Stress Biology, School of Pharmaceutical Sciences, Faculty of Medicine and Life Sciences, Xiamen University, Xiang’an South Road, Xiamen, Fujian 361102, China; Fujian Provincial Key Laboratory of Innovative Drug Target Research, School of Pharmaceutical Sciences, Faculty of Medicine and Life Sciences, Xiamen University, Xiang’an South Road, Xiamen, Fujian 361102, China; State Key Laboratory of Cellular Stress Biology, School of Pharmaceutical Sciences, Faculty of Medicine and Life Sciences, Xiamen University, Xiang’an South Road, Xiamen, Fujian 361102, China; Fujian Provincial Key Laboratory of Innovative Drug Target Research, School of Pharmaceutical Sciences, Faculty of Medicine and Life Sciences, Xiamen University, Xiang’an South Road, Xiamen, Fujian 361102, China; Xiang An Biomedicine Laboratory, School of Pharmaceutical Sciences, Faculty of Medicine and Life Sciences, Xiamen University, Xiang’an South Road, Xiamen, Fujian 361102, China

## Abstract

Protein arginine methyltransferase CARM1 has been shown to methylate a large number of non-histone proteins, and play important roles in gene transcriptional activation, cell cycle progress, and tumorigenesis. However, the critical substrates through which CARM1 exerts its functions remain to be fully characterized. Here, we reported that CARM1 directly interacts with the GATAD2A/2B subunit in the nucleosome remodeling and deacetylase (NuRD) complex, expanding the activities of NuRD to include protein arginine methylation. CARM1 and NuRD bind and activate a large cohort of genes with implications in cell cycle control to facilitate the G1 to S phase transition. This gene activation process requires CARM1 to hypermethylate GATAD2A/2B at a cluster of arginines, which is critical for the recruitment of the NuRD complex. The clinical significance of this gene activation mechanism is underscored by the high expression of CARM1 and NuRD in breast cancers, and the fact that knockdown CARM1 and NuRD inhibits cancer cell growth *in vitro* and tumorigenesis *in vivo*. Targeting CARM1-mediated GATAD2A/2B methylation with CARM1 specific inhibitors potently inhibit breast cancer cell growth *in vitro* and tumorigenesis *in vivo*. These findings reveal a gene activation program that requires arginine methylation established by CARM1 on a key chromatin remodeler, and targeting such methylation might represent a promising therapeutic avenue in the clinic.

## Graphical Abstract

**Graphical Abstract:**
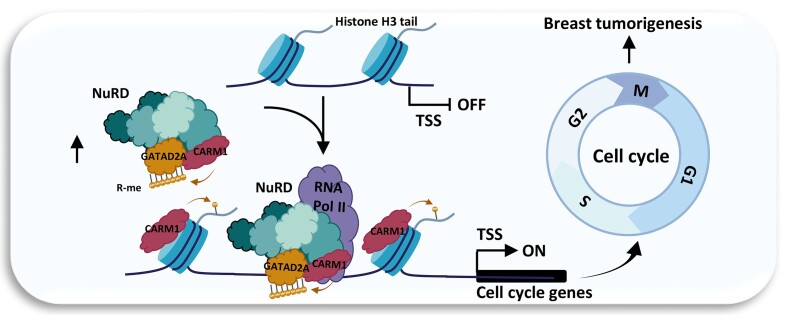

## Introduction

CARM1 (coactivator-associated arginine methyltransferase 1), also known as PRMT4 (protein arginine methyltransferase 4), is a type I arginine methyltransferase catalyzing both mono- and asymmetric di-methylation on arginine residues in proteins, which was originally identified as a binding protein of the p160 coactivators (1). The unique carboxyl-terminus domain of CARM1, which contains a strong autonomous activation activity, collaborates with the methyltransferase activity located at the central portion of CARM1 to mediate its coactivator function (2). To date, CARM1 has been found to methylate histone H3 as well as a large number of non-histone proteins with diverse functions (1,3–6). Transcription factors such as SOX9 (7), PAX7 (8), Notch1 (9), and FOXO3 (10), transcriptional co-factors such as CBP/p300 (11–15), NCOA3 (16), BAF155/SMARCC1 (17), MED12 (18–20), and RNA polymerase II (RNA Pol II) (21), metabolic enzymes such as MDH1 (22) and PKM2 (23), and RNA binding proteins such as PABP1 (24), P54^nrb^ (25), CA150, SmB, U1C and SF3b4 (26) are well-characterized CARM1 substrates. CARM1 has been shown to play a critical role in a myriad of cellular processes including transcription, DNA damage response, pre-mRNA splicing, cell cycle progression, and cellular differentiation. Aberrant expression and/or activation of CARM1 has been linked to a variety of human diseases, particularly cancers, such as breast, prostate, colorectal, lung and liver cancers (27,28). CARM1 is thought to contribute to cancer progression mainly through its coactivator activity targeting a plethora of transcription factors, such as p53, E2F1 and NFκB (29–31), and/or its methyltransferase activity directly targeting oncogenic proteins, such as BAF155, MED12, and PKM2 (17,18,20,23). Among all cancer types, the role of CARM1 in breast cancer was most studied. CARM1 substrates, such as E2F1, BAF155 and MED12, were suggested to be important for CARM1-mediated breast cancer cell growth and tumorigenesis (5,17,18,20,31). Despite the emerging roles it plays in normal and disease contexts, the critical substrates of CARM1 remain to be fully characterized, which hinders our further understanding of the underlying molecular mechanisms on how it exerts its diverse functions.

The NuRD complex (nucleosome remodeling and deacetylase) was first identified as a large multi-subunit complex coupling ATP-dependent chromatin-remodeling and histone deacetylase activities (32–35). It comprises many different subunits, which include ATP-dependent chromatin-remodeling enzymes CHD3 and CHD4, histone chaperones RBBP4 and RBBP7, histone deacetylase HDAC1 and HDAC2, DNA-binding proteins MTA1, MTA2 and MTA3, CpG-binding proteins MBD2 and MBD3, and GATA zinc-finger domain-containing proteins GATAD2A (p66α) and GATAD2B (p66β) (36,37). More recently, it has been shown that KDM1A (LSD1) is also a component of the NuRD complex, expanding NuRD’s chromatin remodeling capacity to include ATPase, histone deacetylase, and histone demethylation activities (38). The homologs or isoforms of some of the subunits in the NuRD complex were found to be mutually exclusive, leading to a horde of NuRD sub-complexes (39,40). The NuRD complex was initially thought to exclusively repress gene transcription due to its composition, such that HDAC1/2 are capable of deacetylating histones and MBDs are connected to DNA methylation (32–34,41). However, recent genome-wide data revealed that the NuRD complex can be localized on promoters and enhancers of genes that are actively being transcribed (40,42–47). The exact role of NuRD in regulating gene expression is not clearly understood, but one hypothesis is that it serves to modulate chromatin structure at active transcriptional sites to fine-tune gene expression (45). Although the change of gene expression by NuRD is moderate, the cumulative effect of this is nevertheless an acute phenotype. Due to its critical roles in regulating basic cellular processes, such as chromatin remodeling, gene transcription, DNA damage repair and cell cycle progression (36,37,48–50), alterations of NuRD activity have been shown to lead to developmental defects, cancers, and accelerated ageing (51–53).

One of the fundamental aspects of understanding the functions of NuRD and the underlying molecular mechanisms is to delineate the NuRD interactome. Efforts have been devoted to investigate the 3D (three dimensional) architecture of the core subunits, aiming to provide insights into the assembly and recruitment of the NuRD complex (36,37). Transcription factors and cofactors, such as GATA1/FOG1, SALL1, c-JUN, IKAROS and ZMYND8, have been shown to recruit the NuRD complex to specific gene loci in specific contexts (40,54–57). Inappropriate localization of the NuRD complex has been suggested to contribute to cancer development (52). Identification of proteins that govern the recruitment of the NuRD complex will shed light on understanding the molecular mechanisms in NuRD-regulated gene transcription.

In the current study, we reported that CARM1 directly interacts the GATAD2A/2B subunit in the NuRD complex. They are localized on the promoter regions of and regulate the transcriptional activation of a large cohort of genes with implications in cell cycle control. CARM1-mediated gene activation requires CARM1-mediated GATAD2A/2B methylation at a stretch of arginine sites. To underscore the biological significance of CARM1-mediated GATAD2A/2B methylation and transcriptional events, high expression of CARM1 and NuRD is observed in clinical breast cancer samples, and knockdown of CARM1 and NuRD significantly attenuates breast cancer cell growth *in vitro* and tumorigenesis *in vivo*.

## Materials and methods

### Plasmids and cloning procedures

CARM1 in pGEX-6P-1 (GE Healthcare, 28954648) and p3xFLAG-CMV-10 (Sigma, E7658) expression vectors were described previously (58). HA-tag was added to the carboxy-terminus of CARM1 when cloned into p3xFLAG-CMV-10 vector (pCMV10-Flag-CARM1-HA). Full-length (FL) CHD4, RBBP4, RBBP7, HDAC1, HDAC2, MTA1, MBD3, GATAD2A and KDM1A, and truncated MTA2 were PCR-amplified from cDNA templates (gifts from Dr Jiahuai Han at Xiamen University) by using Transstart FastPfu Fly DNA Polymerase (TransGen Biotech, AP221-02) and then cloned into p3xFLAG-CMV-10 expression vector. CARM1 enzymatically dead mutant (E267Q) and GATAD2A (7R/K) mutant were generated by using QuikChange Lightning Site-Directed Muta-genesis Kit (Stratagene, 210518). ShRNAs specially targeting *CARM1* or individual subunit in the NuRD complex were cloned into lentiviral pLKO.1 (Addgene, 10878) vector with AgeI (Biolabs, R3552) and EcoRI (Biolabs, R3101).

### SiRNA transfection, RNA isolation and RT-qPCR

SiRNA transfections were performed using Lipofectamine 2000 (Invitrogen, 11668019) according to the manufacturer's protocol. Total RNA was isolated using RNeasy Mini Kit (Qiagen, 74104) following the manufacturer's protocol. First-strand cDNA synthesis from total RNA was carried out using GoScript Reverse Transcription System (Promega, A2800), followed by quantitative PCR (qPCR) using The AriaMx Real-Time PCR system (Agilent Technologies, G8830A). Sequence information for all primers used to check gene expression was presented in Supplementary Table S2. siCARM1-1: 5′-GAUAGAAAUCCCAUUCAAA-3′; siCARM1-2: 5′-GUAACCUCCUGGAUCUGAA-3′; siGATAD2A: 5′- GCGGCAGAGUCAAAUACAA-3′; siCHD4: 5′-GCAUGUCCUUACUAGAAUU-3′; siKDM1A: 5′- CUGGAAAUGACUATGAUUUAA-3′; siHDAC1: 5′-GGCAAGUAUUAUGCUGUUA-3′; siHDAC2: 5′- UCCGUAAUGUUGCUCGAUG -3′.

### Plasmids transfection, lentiviral vectors packaging and infection

Plasmid transfections were performed using Polyethyleneimine (PEI, Polysciences, 00618-25) according to the manufacturer's protocol. Lentiviral vectors packaging and infection: HEK293 cells were seeded in culture plates coated with poly-d-lysine (0.1% (w/v), Sigma, P7280) and transfected with lentiviral vectors together with packaging vectors, pMDL, VSVG and REV, at a ratio of 10:5:3:2 using Polyethyleneimine (PEI, Polysciences, 00618-25) for 48 h according to the manufacturer's protocol. Virus was collected, filtered and added to MCF7 cells in the presence of 10 μg/ml polybrene (Sigma, H9268). shCARM1-1: 5′-GATAGAAATCCCATTCAAA-3′; shCARM1-2: 5′-GTAACCTCCTGGATCTGAA-3′; shGATAD2A: 5′- GCGGCAGAGTCAAATACAA-3′; shGATAD2A-3UTR: 5′-GCCTTCCCATGGCGATCTATA-3′.

### Immunoblotting and immunoprecipitation

Immunoblotting and immunoprecipitation were performed following the protocol described previously (59,60). Antibodies used in the current study was presented in Supplementary Table S3.

### Purification of CARM1-associated proteins

HEK293 cells were transfected with pCMV10-Flag-CARM1-HA, and then selected with G418 (1 mg/ml) (Gibco, 10131035). Resultant single colonies were subjected to immunoblotting to examine the expression of exogenous CARM1 protein. To purify proteins associated with CARM1, cytosolic and nuclear fractions were first separated and nuclear extracts were then prepared in a buffer containing 50 mM Tris–HCl (pH 7.4), 150 mM NaCl, 1 mM EDTA and 1% Triton X-100, followed by affinity purification using Anti-FLAG M2 Affinity gel (Millipore, A2220). DNase I (Promega, M6101) was added during purification to remove genomic DNA. Immunoprecipitates were then washed with low-salt buffer containing 50 mM Tris–HCl (pH 7.4), 150 mM NaCl, 1 mM EDTA and 1% Triton X-100 for three times followed by high-salt buffer containing 50 mM Tris–HCl (pH 7.4), 420 mM NaCl, 1 mM EDTA and 1% Triton X-100 for two times before elution with 3× Flag peptides (Sigma, F4799). The eluates were subjected to in solution digestion and LC–MS/MS analysis directly or resolved by SDS-PAGE gel, silver-stained and then subjected to in gel digestion following the protocol described below.

### Generation of CARM1 knockout cell lines using CRISPR/Cas9 system

CARM1 knock out (KO) HEK293 cells were generated by using CRISPR/Cas9 system. Specifically, gRNA sequence (5′-CACCGATCATCATCTCGGAGCCCAT-3′) targeting CARM1 was first cloned into gRNA cloning vector (Addgene, 41824) and confirmed by sequencing. HEK293 cells were then transfected with pcDNA3.3-hCas9 (Addgene, 41815) and gRNA expression vectors, followed by G418 (1 mg/ml) (Gibco, 10131035) selection. Single colonies were subjected to immunoblotting to select the ones with CARM1 knockout, which were further validated by Sanger sequencing of PCR products using primer set spanning gRNA targeting region and genomic DNA as template. The primer set was as follows: Forward (F) 5′-TCGTGGTCATCCCGGGCA-3′ and Reverse (R) 5′-GTAGCTCTCCAGCATGCG-3′.

### Chromatin immunoprecipitation (ChIP), chromatin immunoprecipitation coupled with high throughput sequencing (ChIP-Seq) and computational analysis of ChIP-Seq data

For ChIP assays, cells were fixed with 1% formaldehyde (Sigma, F8775) for 15 minutes (mins) at room temperature (RT) (for RNA Pol II (Bethyl Laboratory A300-653A) ChIP) or fixed with disuccinimidyl glutarate (DSG) (2 mM) (Proteochem, C1104) for 45 mins at RT, washed twice with PBS and then double-fixed with 1% formaldehyde for another 15 min at RT (for HA-tagged proteins (Abcam, ab9110), CARM1 (CST, 12495), CHD4 (Abcam, ab70469), HDAC2 (Santa Cruz Biotechnology, sc7899), GATAD2A (Abcam, ab87663 or Proteintech, 12294-1-AP), and KDM1A (Bethyl Laboratory, A300-215A)). Fixation was stopped by adding glycine (0.125 M) and incubating for 5 min at RT, followed by washing with PBS twice. Chromatin DNA was sheared to 300–500 bp average in size through sonication. Resultant was immunoprecipitated with control IgG or specific antibodies overnight at 4°C, followed by incubation with protein G magnetic beads (Biorad, 161-4023) for an additional 2 h. After washing and elution, the cross-linked protein-DNA complex was reversed by heating at 65°C overnight. Immunoprecipitated DNA was purified by using QIAquick spin columns (Qiagen, 28115) and subjected to either qPCR analysis or high throughput sequencing.

ChIP-seq sample preparation and computational analysis of ChIP-seq data were performed as following.

Library construction: the libraries were constructed following Illumina ChIP-seq Sample prep kit (Illumina, 11257047 A). Briefly, ChIP DNA was end-blunted and added with an ‘A’ base so the adaptors from Illumina with a ‘T’ can ligate on the ends. Then 200–400 bp fragments are gel-isolated and purified. The library was amplified by 18 cycles of PCR.

Primary analysis of ChIP-Seq datasets: the image analysis and base calling were performed by using Illumina's Genome Analysis pipeline. The sequencing reads were aligned to hg19 Refseq database by using Bowtie2 (http://bowtie-bio.sourceforge.net/bowtie2/index.shtml) (61) with default parameters. Both uniquely aligned reads and reads that align to repetitive regions were kept for downstream analysis (if a read was aligned to multiple genomic locations, only one location with the best score was chosen). Clonal amplification was circumvented by allowing maximal one tag for each unique genomic position. The identification of ChIP-seq peaks was performed using HOMER (http://biowhat.ucsd.edu/homer) (62). The threshold for the number of tags that determined a valid peak was selected at a false discovery rate (FDR) of 0.001. Fourfold more tags relative to the local background region (10 kb) were also required to avoid identifying regions with genomic duplications or non-localized binding. Genomic distribution was done by using the default parameters from HOMER with minor modifications, in which promoter peaks were defined as those with peak center falling between 1000 bp downstream and 5000 bp upstream of transcript start sites (TSSs). Motif analysis was performed using HOMER. Tag density for histograms (50 bp/bin), box plots and heat maps were generated by using HOMER or deep Tools (https://deeptools.readthedocs.io/en/develop) (63). Box plots were then generated by R software (https://www.r-project.org/) and significance was determined using Student's t test. Heat maps were visualized using Java TreeView (http://jtreeview.sourceforge.net) (64) or deep Tools.

ChIP-seq files were deposited in the Gene Expression Omnibus database under accession GSE209910. The following link has been created to allow review of record GSE209910 while it remains in private status: https://www.ncbi.nlm.nih.gov/geo/query/acc.cgi?acc=GSE209910 (token: gzudaioqxxirzwx).

### RNA sequencing (RNA-seq) and computational analysis of RNA-seq data

Total RNA was isolated using RNeasy Mini Kit (Qiagen, 74104) following the manufacturer's protocol. DNase I (Qiagen, 79254) in column digestion was included to ensure the RNA quality. RNA library preparation was performed by using NEBNext® Ultra™ Directional RNA Library Prep Kit for Illumina (Illumina, E7420L). Paired-end sequencing was performed with Illumina HiSeq platform at RiboBio Co., Ltd or Amogene Biotech Co., Ltd.

Sequencing reads were aligned to hg19 Refseq database by using Tophat (65). Both uniquely aligned reads and the sequencing reads that aligned to repetitive regions were kept for downstream analysis (if a read aligned to multiple genomic locations, only one location with the best score was chosen). Only reads on exons were counted for quantifying gene expression by HOMER. DESeq2 was used to compute the significance of differential expressed genes (FDR ≤ 0.05) (66). Only coding genes with FPKM larger than 0.5, either in control or siRNA-treated sample, were included in our analysis. FPKM of a gene was calculated as mapped reads on exons divided by exonic length and the total number of mapped reads. Box plots were then generated by R software and significance was determined using Student's *t* test. Heat maps were visualized using Java TreeView or deepTools.

RNA-seq files were deposited in the Gene Expression Omnibus database under accession GSE209910. The following link has been created to allow review of record GSE209910 while it remains in private status: https://www.ncbi.nlm.nih.gov/geo/query/acc.cgi?acc=GSE209910 (token: gzudaioqxxirzwx).

### Protein purification from bacterial cells or HEK293 cells

GST-tagged proteins were expressed in BL21 (DE3) bacterial cells (Stratagene, EC0114) and purified by using Glutathione agarose (Millipore, G4520), following the protocol described previously (59,60). Flag–tagged proteins were expressed in HEK293 cells and cells were lysed in lysis buffer containing 50 mM Tris–HCl, pH 7.4, 150 mM NaCl, 1 mM EDTA, 1% Triton X-100. Flag-tagged proteins were then affinity-purified by using Anti-FLAG M2 Affinity gel (Millipore, A2220) and washed extensively with washing buffer containing 50 mM Tris–HCl, pH 7.4, 420 mM NaCl, 1 mM EDTA, 1% Triton X-100 before elution with 3× Flag peptides (Sigma, F4799).

### GST pull-down assay

*In vitro* purified, bacterially-expressed GST-tagged proteins were incubated with Flag-tagged proteins purified from over-expressed HEK293 cells at 4°C for at least 4 h. Glutathione agarose (Millipore, G4520) were then washed three times with washing buffer containing 300 mM KCl and 0.05% NP-40 before loading onto SDS-PAGE gel and immunoblotting.

### *In vitro* methylation assay

*In vitro* methylation assay was performed in methylation buffer (50 mM Tris–HCl, pH 8.0, 20 mM KCl, 5 mM DTT, 4 mM EDTA) in the presence of 1 μCi l-[methyl-^3^H]-methionine (PerkinElmer, NET061) at 37 °C for 1 h. The reaction was stopped by adding SDS sample buffer followed by SDS-PAGE gel and autoradiogram.

### Stable isotope labeling by amino acids in cell culture (SILAC), affinity purification, in solution digestion and LC–MS/MS analysis

Wild-type (WT) and CARM1 knockout (KO) HEK293 cells were grown in SILAC DMEM (Invitrogen, 88364) supplemented with l-lysine/arginine and l-lysine/arginine-U-13C6 (Cambridge Isotope Laboratories, 1119-34-2, 201740-91-2, 657-26-1, 2024-06-540), respectively, together with 10% dialyzed FBS (Gibco, 30067334), l-glutamine (Gibco, A2916801) and penicillin/streptomycin (Gibco, 15140122) for 2 weeks followed by transfected with vectors expressing pCMV10-3xFlag-GATAD2A for 48 h. Cells were then lysed in a buffer containing 50 mM Tris–HCl (pH 7.4), 150 mM NaCl, 1 mM EDTA and 1% Triton X-100, pooled and subjected to affinity purification by using Anti-FLAG M2 Affinity gel (Millipore, A2220), washed extensively with a buffer containing 50 mM Tris–HCl (pH 7.4), 150 mM NaCl, 1 mM EDTA and 1% Triton X-100, and eluted with 3× Flag peptides (Sigma, F4799). Eluates were then subjected to in solution digestion following the protocol as described previously (20). MS experiments were performed on a nanoscale UHPLC system (Thermo Fisher Scientific, EASY-nLC1200) connected to an Orbitrap Fusion Lumos equipped with a nanoelectrospray source (Thermo Fisher Scientific, IQLAAEGAAPFADBMBHQ). The peptides were separated on a RP-HPLC analytical column (75 μm × 25 cm) packed with 2 μm C18 beads (Thermo Fisher Scientific, 164941) using a linear gradient ranging from 9% to 28% ACN in 90 mins and followed by a linear increase to 45% B in 20 min at a flow rate of 300 nl/min. The Orbitrap Fusion Lumos acquired data in a data-dependent manner alternating between full-scan MS and MS2 scans. The MS spectra (350–1500 *m*/*z*) were collected with 120000 resolution, AGC of 4 × 10^5^, and 50 ms maximal injection time. Selected ions were sequentially fragmented in a 3 s cycle by HCD with 30% normalized collision energy, specified isolated windows 1.6 *m*/*z*, 30 000 resolution. AGC of 5 × 10^4^ and 120 ms maximal injection time were used. Dynamic exclusion was set to 15 s. Raw data was processed using Proteome Discoverer (PD, version 2.1), and MS/MS spectra were searched against the reviewed Swiss-Prot human proteome database. The abundance of peptides was specified by PD 2.1, in which the minimum and maximum value was set to 0 and 200, respectively. For all methylated peptides in GATAD2A detected, the abundance changed from 200 to 0, indicating that methylation detected on these peptides in WT cells were abolished in CARM1 KO cells.

### Mining of the cancer genome atlas (TCGA) data and Kaplan–Meier survival analysis

Expression data (RPKM) of CARM1 and GATAD2A in a cohort of TCGA clinical breast samples (tumor: 1102; normal: 113) was downloaded from GDC Data Portal. Box plots were generated by R software and significance was determined using Student's t-test. Heat maps were visualized using Java TreeView. Kaplan–Meier survival analyses for OS (overall survival) (*n* = 1402) of breast cancer patients were done following the link below: http://kmplot.com/analysis/index.php?p=service&default=true.

### Cell proliferation assay, FACS (fluorescence-activated cell sorting) analysis, colony formation assay and tumor xenograft assay

Cell viability was measured by using a CellTiter 96 AQueous one solution cell proliferation assay kit (Promega, G3580) following the manufacturer's protocol. Briefly, cells were seeded in culture plates coated with poly-d-lysine (0.1% (w/v), Sigma, P7280) and transfected with siRNA for different time points followed by cell proliferation assay. To measure cell viability, 20 μl of CellTiter 96 AQueous one solution reagent was added per 100 μl of culture medium, and the culture plates were incubated for 1 hr at 37 °C in a humidified, 5% CO_2_ atmosphere incubator. The reaction was stopped by adding 25 μl of 10% SDS. Data was recorded at wavelength 490 nm using a Thermo Multiskan MK3 Microplate Reader.

For FACS analysis, cells were trypsinized, washed with PBS and fixed with ethanol at 4 °C overnight. Cells were then washed with PBS and stained with PI/Triton X-100 staining solution (0.1% (v/v) Triton X-100, 0.2 mg/ml DNase-free RNase A (Sigma, R4642), 0.02 mg/ml propidium iodide (Roche, 11348639001)) at 37°C for 15 min. DNA content was then measured and about 10^5^ events were analyzed for each sample. Data were analyzed using ModFit LT (Verity Software House).

For colony formation assays, 2000 cells transfected with siRNA, were maintained in a 6-well plate, and colonies were examined 10 days after. Briefly, colonies were fixed with methanol/acid solution (3:1) for 5 min and stained with 0.1% crystal violet for 15 min. For quantification, the crystal violet dye was released into 10% acetic acid and measured at wavelength 590 nm (OD590).

For tumor xenograft assay, female BALB/C nude mice (age 4–6 weeks) were subcutaneously implanted with 5 × 10^6^ of MCF7 cells or MDA-MB-231 cells suspended in DMEM medium without FBS. To promote MCF7 cell tumorigenicity, each nude mouse was brushed with estrogen (E_2_, 10^−2^ M) (Sigma, E2758) every 3 days for the duration of the experiments. All mice were euthanized 6 weeks after subcutaneous injection. Tumors were then excised, photographed and weighted. For EZM2302 treatment in *vivo*, when tumor size reached approximately 100 mm^3^, mice were randomly assigned into three groups and treated with EZM2302 intraperitoneally every 2 days. Tumors were then excised, photographed and weighted. All animals were housed in the Animal Facility at Xiamen University under pathogen-free conditions, following the protocol approved by the Xiamen Animal Care and Use Committee.

## Results

### CARM1 interacts with the NuRD complex

**Figure 1.**
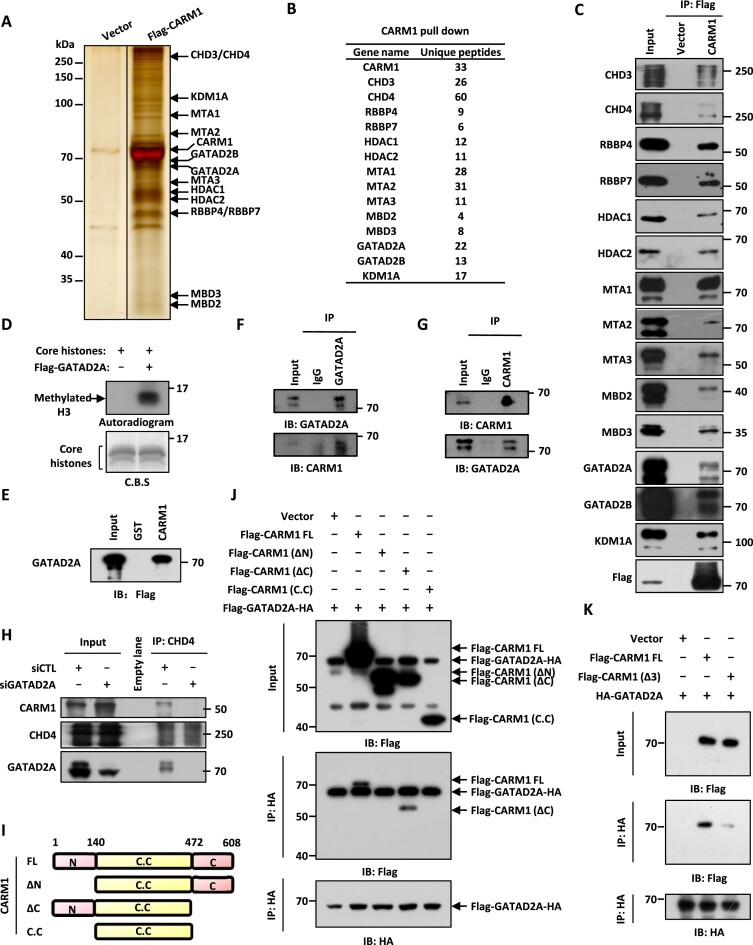
CARM1 interacts with the NuRD complex. (**A**) HEK293 cells stably expressing Flag-HA-tagged CARM1 or empty vector were subjected to affinity purification, and the eluates were resolved by SDS-PAGE gel, silver-stained and subjected to mass spectrometry analysis. (**B**) Subunits in the NuRD complex and the corresponding number of unique peptides identified are shown as indicated. (**C**) HEK293 cells as described in (A) were subjected to immunoprecipitation (IP) with anti-Flag antibody followed by immunoblotting (IB) with antibodies as indicated. (**D**) *In vitro* methylation assay was performed by mixing core histones with eluates from control vector or Flag-tagged GATAD2A affinity purification in HEK293 cells, followed by autoradiogram (upper panel). Loading of core histones is shown by coomassie blue staining (CBS) (bottom panel). (**E**) GST pull-down assay was performed by mixing Flag-tagged GATAD2A purified from over-expressed HEK293 cells with GST or GST-tagged CARM1 followed by immunoblotting (IB) with anti-Flag antibody. (**F, G**) HEK293 cells were subjected to co-immunoprecipitation (Co-IP) assay with control IgG, anti-GATAD2A (F), or anti-CARM1 (G) antibody, followed by immunoblotting (IB) analysis with antibodies as indicated. (**H**) HEK293 cells transfected with control siRNA (siCTL) or siRNA specifically targeting GATAD2A (siGATAD2A) were subjected to immunoprecipitation (IP) with anti-CHD4 antibody, followed by immunoblotting (IB) with antibodies as indicated. (**I**) Schematic representation of full length (FL) and truncated CARM1 proteins. ΔN: amino (N)-terminus deletion; ΔC: carboxyl (C)-terminus deletion; C.C: catalytic core. (**J**) HEK293 cells were transfected with vectors expressing Flag and HA-tagged GATAD2A and empty vector, full length (FL), amino (N)-terminal deletion (ΔN), carboxyl (C)-terminal deletion (ΔC) or catalytic core only (C.C) CARM1 followed by immunoprecipitation (IP) with anti-HA antibody and immunoblotting (IB) with anti-Flag or anti-HA antibody. (**K**) HEK293 cells were transfected with vectors expressing HA-tagged GATAD2A and empty vector, full length (FL), or region 3-deleted (Δ3) CARM1 followed by immunoprecipitation (IP) with anti-HA antibody and immunoblotting (IB) with anti-Flag or anti-HA antibody.

Our previous study demonstrated that CARM1 methylates a large number of non-histone proteins (*n* = 301) with implications in a plethora of cellular processes (6). To further study the function of CARM1-mediated methylation, we first sought to identify CARM1-associated proteins as which might help us to better define its ‘true’ substrates. HEK293 cells stably expressing CARM1 were subjected to affinity purification followed by mass spectrometry analysis (Supplementary Figure S1A). As previously reported, the SWI/SNF chromatin remodeling complex was found to be abundantly present in proteins associated with CARM1 (17,67) (Supplementary Figure S1B). Unexpectedly, all the subunits in a second chromatin remodeling complex, the NuRD complex, were also abundantly present, which included the ATPase CHD3/4, histone chaperones RBBP4/7, histone deacetylase HDAC1/2, DNA-binding proteins MTA1/2/3, CpG-binding proteins MBD2/3, zinc-finger proteins GATAD2A/2B and histone demethylase KDM1A (Figure 1A and B). When we compared CARM1-associated proteins (*n* = 4805) with CARM1-methylated proteins (*n* = 301) we reported previously (6), there were 199 proteins were found to be in common (Supplementary Figure S1C). GATAD2A/2B in the NuRD complex was among these 199 proteins. The presence of the entire NuRD complex in the list of CARM1-associated proteins as well as GATAD2A/2B in the list of CARM1-methylated proteins caught our attention.

The interaction between CARM1 and each subunit in the NuRD complex or representative subunits in the SWI/SNF complex was confirmed (Figure 1C and Supplementary Figure S1D). Similarly, subunits in the NuRD complex were similarly pulled down by CARM1 when we used anti-HA antibody for immunoprecipitation (Supplementary Figure S1E). To further support the interaction between CARM1 and NuRD, eluates from GATAD2A affinity purification exhibited methyltransferase activity towards histone H3, the histone substrate of CARM1 (Figure 1D).

Next, we examined which subunit in the NuRD complex interacts with CARM1 directly. *In vitro* GST pull-down assay was performed by mixing purified subunit in the NuRD complex including CHD4 (representing CHD3/4), RBBP4 (representing RBBP4/7), HDAC1 (representing HDAC1/2), MTA1 (representing MTA1/2/3), MBD3 (representing MBD2/3), GATAD2A (representing GATAD2A/2B) or KDM1A with CARM1. The results revealed that CARM1 specifically interacted with GATAD2A (Figure 1E, and Supplementary Figure S1F and G). The interaction between CARM1 and GATAD2A were also confirmed at endogenous level in HEK293 cells through co-immunoprecipitation analysis (Figure 1F and G). We then knocked down GATAD2A in HEK293 cells followed by immunoprecipitation with anti-CHD4 antibody to pull down the NuRD complex, and found that the interaction between CARM1 and the NuRD complex was significantly attenuated compared to control cells, supporting the notion that CARM1 interacts with NuRD through GATAD2A (Figure 1H). Quantitative MS analysis confirmed that CARM1 association with NuRD complex was attenuated (Supplementary Figure S1H). Meanwhile, the integrity of the NuRD complex was jeopardized when GATAD2A was knocked down (Supplementary Figure S1H).

To determine which domain in CARM1 is required for its interaction with GATAD2A, we transfected HEK293 cells with vectors expressing GATAD2A and full length (FL), amino (N)-terminal deleted (ΔN), carboxyl (C)-terminal deleted (ΔC), or catalytic core only (C.C)-CARM1 followed by immunoprecipitation and immunoblotting. CARM1 N-terminus was found to be required for its interaction with GATAD2A (Figure 1I and J). The GATAD2A-interacting region in CARM1 N-terminus was further narrowed down to amino acid 63–88 (referred as region 3) by GST pull-down assay (Supplementary Figure S1I–K). To further confirm that region 3 was required for CARM1 to interact with GATAD2A, purified full length (FL) or region 3-deleted (Δ3) CARM1 was mixed with GATAD2A or MED12 C-terminus (aa 1616–2177), which was reported to interact with CARM1 (5). Deletion of region 3 nearly abolished the interaction between CARM1 and GATAD2A, whereas it had no effects on MED12 C-terminus (Figure 1K and Supplementary Figure S1L). Taken together, our data suggested that CARM1 interacts with NuRD through GATAD2A, and presumably GATAD2B too, due to high homology.

### CARM1 hypermethylates GATAD2A/2B in the NuRD complex

**Figure 2.**
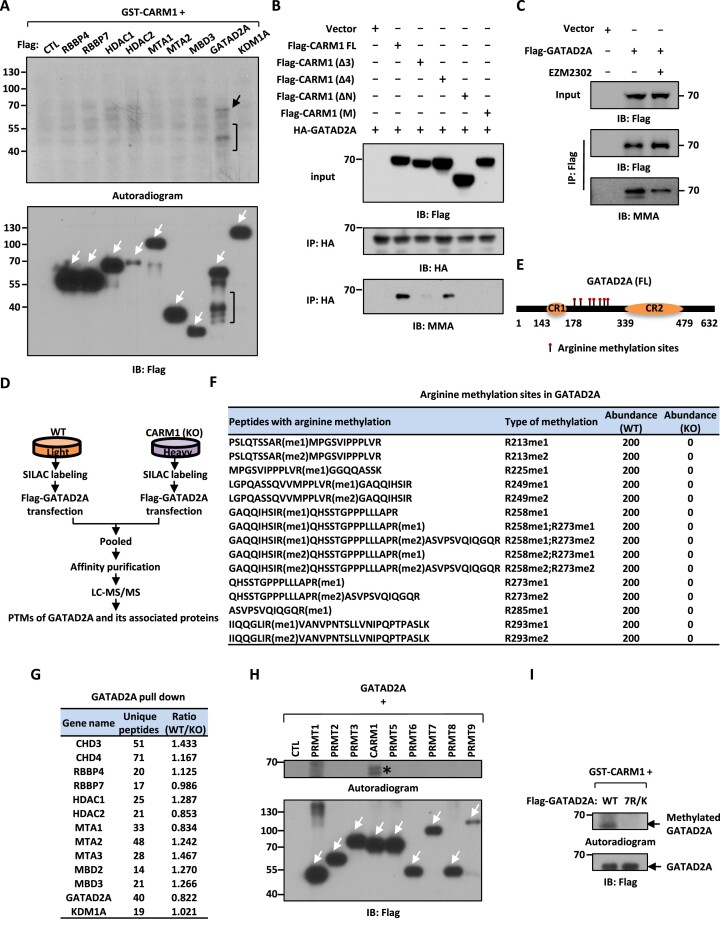
CARM1 hypermethylates GATAD2A/2B in the NuRD complex. (**A**) *In vitro* methylation assay was performed by mixing GST-tagged CARM1 purified from bacterial cells with Flag-tagged, representative subunits in the NuRD complex including RBBP4, RBBP7, HDAC1, HDAC2, MTA1, MTA2 (truncation), MBD3, GATAD2A and KDM1A purified from over-expressed HEK293 cells, followed by autoradiogram to examine CARM1-mediated methylation (upper panel) or immunoblotting (IB) using anti-Flag antibody to examine the expression of the NuRD subunits (bottom panel). Black arrow and bracket (upper panel) indicated methylation of GATAD2A and its proteolytic fragments, respectively. White arrows indicated the size of the corresponding subunits in the NuRD complex. (**B**) *In vitro* methylation assay was performed by mixing purified GATAD2A with full length (FL), region 3-deleted (Δ3), region 4 deleted-(Δ4), amino (N)-terminal-deleted (ΔN), or enzymatic dead (M) CARM1, followed by immunoblotting (IB) with antibodies as indicated. (**C**) HEK293 cells were transfected with or without Flag-tagged GATAD2A and treated with or without EZM2302 (50 μM, 48 h), followed by immunoprecipitation (IP) with anti-Flag antibody and immunoblotting (IB) analysis with antibodies as indicated. (**D**) Schematic representation of the protocol applied for detecting differential binding proteins and post-translational modifications (PTMs) of GATAD2A in WT and CARM1 (KO) HEK293 cells. WT and CARM1 (KO) HEK293 cells were subjected to SILAC labeling and then transfected with vectors expressing Flag-tagged GATAD2A. Cells were then lysed, pooled and subjected to affinity purification using M2 agarose followed by mass spectrometry (MS) analysis. (**E**) Schematic representation of domain architecture of GATAD2A. Arginine methylation sites are shown by matchsticks. CR: Coil-coil region; FL: full-length. (**F**) Arginine methylation sites identified in GATAD2A through mass spectrometry analysis as shown in (C). me1: mono-methylation; me2: di-methylation. (**G**) List of subunits in the CARM1 and NuRD complex identified to be associated with GATAD2A by mass spectrometry (MS) analysis, and the number of peptides and the ratio of the abundance of each subunit in WT and CARM1 (KO) cells are shown. (**H**) *In vitro* methylation assay was performed by mixing Flag-tagged GATAD2A with PRMT proteins purified from over-expressed HEK293 cells as indicated, followed by autoradiogram (upper panel) or immunoblotting (IB) using anti-Flag antibody (bottom panel). White arrows indicated the expression of all PRMTs. Star indicated methylation of GATAD2A. (**I**) *In vitro* methylation assay was performed by mixing GST-tagged CARM1 purified from bacterial cells with Flag-tagged wild-type (WT) or GATAD2A mutant with seven arginine methylation sites substituted with lysines (7R/K) purified from over-expressed HEK293 cells, followed by autoradiogram (upper panel) or immunoblotting (IB) using anti-Flag antibody (bottom panel).

Having demonstrated that CARM1 interacts with GATAD2A/2B in the NuRD complex, we next sought to fully map the arginine methylation sites in GATAD2A/2B in order to understand the function of CARM1-mediated methylation. In our previous report, GATAD2A and GATAD2B in the NuRD complex were found to be exclusively methylated by CARM1 (i.e. methylation signals found on all arginine sites in GATAD2A/2B were completely abolished when CARM1 was depleted in cells.) (Supplementary Figure S2A and B) (6). Due to the high sequence homology between GATAD2A and GATAD2B in the region found to be methylated by CARM1, we focused on GATAD2A only to examine CARM1-mediated methylation. To validate GATAD2A methylation by CARM1, in *vitro* methylation assay was performed by mixing individual purified NuRD subunits with recombinant GST-CARM1 proteins. CARM1 was found to predominantly methylate GATAD2A out of all the subunits tested (Figure 2A), which was consistent with the observation that GATAD2A specifically interacts with CARM1 (Figure 1E). Collaborating with the finding that region 3 in CARM1 was required for CARM1 to target GATAD2A, deletion of region 3 (Δ3) nearly abolished CARM1-mediated GATAD2A methylation (Figure 2B). As expected, N-terminal-deleted (ΔN) and enzymatically dead (M) CARM1 lost their ability to methylate GATAD2A (Figure 2B). Serving as a negative control, deletion of region 4 (Δ4) exhibited no impact on GATAD2A methylation (Figure 2B). Furthermore, deletion of region 3 had no impact on CARM1-mediated methylation on core histones/histone H3 or MED12 (Supplementary Figure S2C and D), indicating that although CARM1 utilizes its N-terminus to target multiple substrates, the specific regions mediating these interactions appear to be different. To support that CARM1 methylates GATAD2A, GATAD2A methylation was found to be inhibited by EZM2302, a selective inhibitor of CARM1 (Figure 2C).

To further fully uncover the arginine methylation sites in GATAD2A and their regulation by CARM1, wild-type or CARM1 knockout cells were subjected to SILAC labeling, transfected with Flag-tagged GATAD2A, pooled and followed by affinity purification and mass spectrometry analysis (Figure 2D and Supplementary Figure S2E). GATAD2A was found to be hypermethylated at seven arginine sites clustered in the region between the coil-coil domain (CR1) and the C-terminal GATA-like zinc finger domain (CR2), which were mono- and di-methylated arginine 213 (R213me1/2), R225me1, R249me1/2, R258me1/2, R273me1/2, R285me1 and R293me1/2 (Figure 2E, F, Supplementary Figure S2F and Supplementary Table S1). Importantly, the methylation on all arginine sites was abolished in CARM1 knockout cells, further supporting that GATAD2A was exclusively methylated by CARM1 in cells (Figure 2F). It should be noted that the composition of the NuRD complex was not significantly altered when CARM1 was depleted based on our mass spectrometry analysis (Figure 2G). To confirm that GATAD2A is exclusively methylated by CARM1 *in vitro*, *in vitro* methylation assay was performed by mixing purified GATAD2A with each individual PRMT, PRMT1 to 9. It was found that CARM1 uniquely methylated GATAD2A (Figure 2H). The activity of all PRMTs was shown by using core histone as substrates here and in our previous reports (Supplementary Figure S2G) (58). When we mutated all seven arginine sites identified above to lysine in GATAD2A (GATAD2A (7R/K)), the methylation by CARM1 was nearly abolished (Figure 2I). Taken together, GATAD2A in the NuRD complex was specifically and exclusively methylated by CARM1 at a cluster of arginine sites.

### CARM1 and NuRD complex occupy a large number of chromatin sites in common

**Figure 3.**
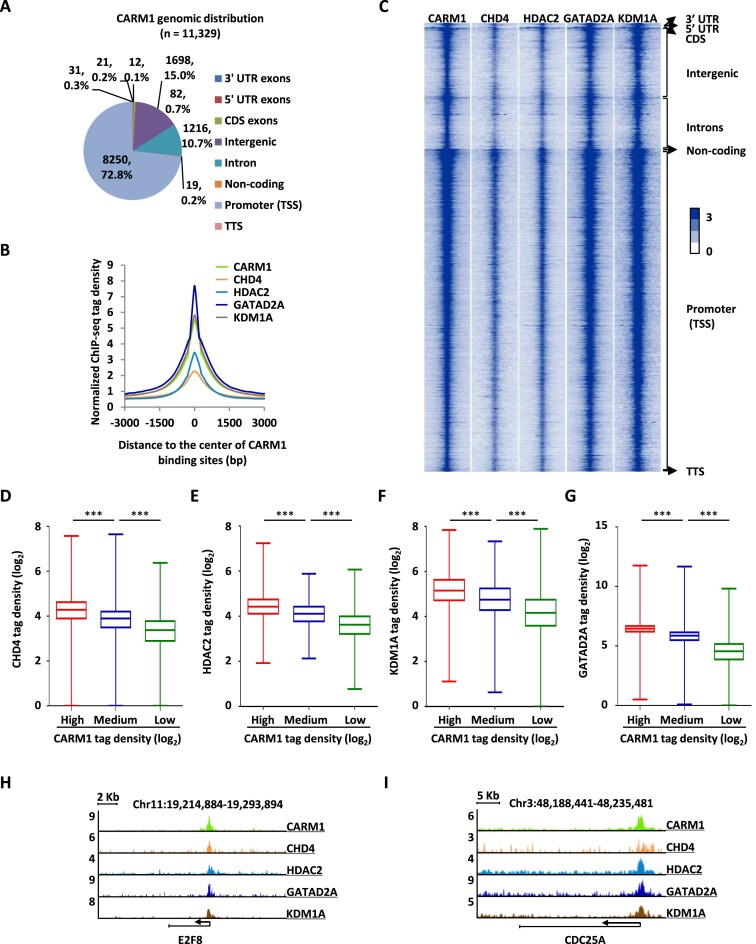
CARM1 and NuRD complex occupy a large number of chromatin sites in common. (**A**) Genomic distribution of CARM1 binding sites identified by ChIP-seq. (**B, C**) Histogram (B) and heat map (C) representation of CARM1, CHD4, HDAC2, GATAD2A and KDM1A ChIP-seq tag density centered on CARM1 binding sites. bp: base pair. (**D–G**) Box plot representation of ChIP tag density (log_2_) of CHD4 (D), HDAC2 (E), KDM1A (F) and GATAD2A (G) on CARM1 binding sites, which were divided into three sub-classes, high, medium and low based on ChIP-seq tag density (± s.e.m., ***P < 0.001). (**H, I**) Genome browser views of CARM1, CHD4, HDAC2, GATAD2A and KDM1A ChIP-seq on *E2F8* (H) and *CDC25A* (I) genes.

The observation that CARM1 interacts and methylates the NuRD complex prompted us to examine the biological significance of this connection between CARM1 and the NuRD complex. Both CARM1 and NuRD have been reported to bind to chromatin to regulate gene transcription. We first tested whether CARM1 and NuRD co-localize on chromatin. ChIP-seq for CARM1 and representative subunits in NuRD including CHD4, HDAC2, GATAD2A and KDM1A was performed in HEK293 cells. A large number of CARM1 binding sites were detected, the majority of which were found to be localized at gene promoter regions (Figure 3A). We then compared the binding sites of CHD4, HDAC2, GATAD2A and KDM1A detected from ChIP-seq to that of CARM1. Tag density plot and heat map demonstrated that CHD4, HDAC2, GATAD2A and KDM1A largely bound to the same genomic region as CARM1 (Figure 3B and C). ChIP-seq tag density of CHD4, HDAC2, GATAD2A and KDM1A was highly correlated to that of CARM1 (Figure 3D–G and Supplementary Figure S3A–D). Binding of CARM1, CHD4, HDAC2, GATAD2A and KDM1A detected from ChIP-seq on representative genes was shown as indicated (Figure 3H, I and Supplementary Figure S3E–H). Taken together, CARM1 and NuRD occupy a large number of promoter sites on chromatin in common.

### CARM1 and NuRD activate a large set of cell cycle genes to promote cell cycle progression in a CARM1 enzymatic activity-dependent manner

**Figure 4.**
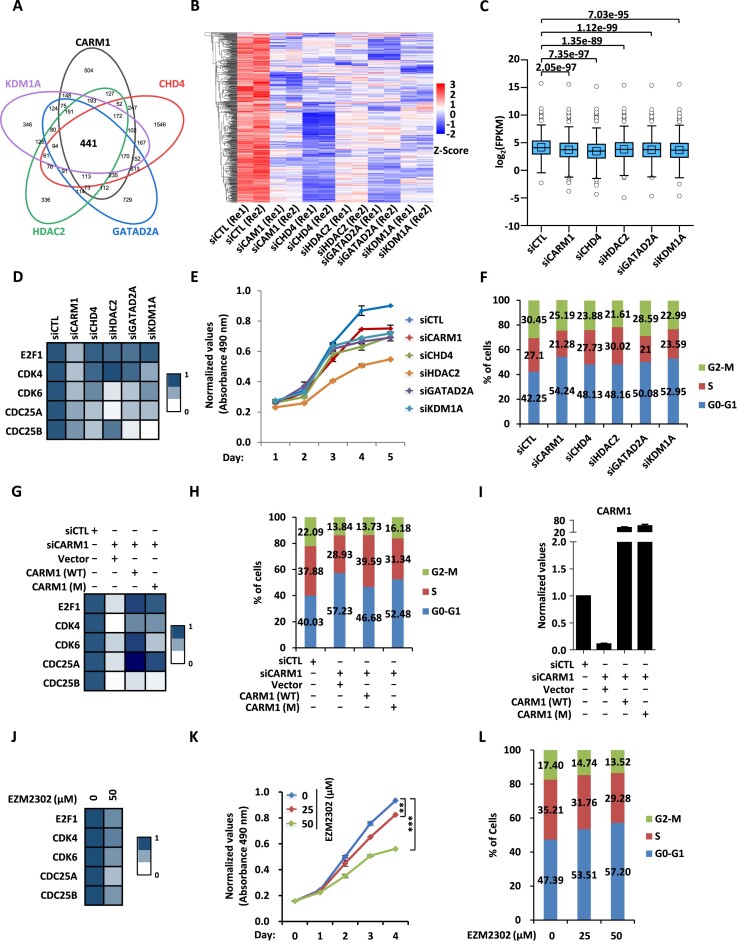
CARM1 and NuRD activate a large set of cell cycle genes to promote cell cycle progression in a CARM1 enzymatic activity-dependent manner. (**A**) HEK293 cells were transfected with control siRNA (*siCTL*) or siRNA specific against *CARM1* (si*CARM1*) or representative subunits in NuRD including *CHD4*, *HDAC2*, *GATAD2A* and *KDM1A* (si*CHD4*, si*HDAC2*, si*GATAD2A* and si*KDM1A*) followed by RNA-seq analysis. Genes positively-regulated by CARM1, CHD4, HDAC2, GATAD2A, and KDM1A in common are shown by venn diagram (*q* < 0.05). (**B, C**) The expression levels (FPKM, log_2_) for those 441 genes as described in (A) are shown by heat map (B) and box plot (C). (**D**) HEK293 cells as described in (A) were subjected to RT-qPCR analysis to examine the expression of selected cell cycle genes as indicated, and data was represented by heat map. (**E, F**) HEK293 cells as described in (A) were subjected to cell proliferation assay (E) and FACS analysis (F). (**G–I**) HEK293 cells were transfected with control siRNA (*siCTL*) or siRNA specifically targeting *CARM1* (si*CARM1*) in the presence or absence of control vector or vector expressing wild-type CARM1 (WT) or its enzymatically deficient mutant (M), followed by RT-qPCR analysis to examine the expression of selected cell cycle genes as indicated (G) and CARM1 (I), and FACS analysis to check cell cycle progression (H). (**J**) HEK293 cells treated with or without EZM2302 (50 μM, 48 h) were subjected to RNA extraction and RT-qPCR analysis to examine the expression of selected cell cycle genes as indicated. (**K, L**) HEK293 cells treated with or without EZM2302 at concentration as indicated were subjected to cell proliferation assay (K) and FACS analysis (L) (± s.e.m., **P < 0.01, ***P < 0.001).

The fact that CARM1 and NuRD bind to a large set of gene promoter regions in common prompted us to examine whether they regulate the expression of these genes. To this end, HEK293 cells were transfected with control siRNA or siRNA specifically targeting *CARM1* or representative subunits in the NuRD complex including *CHD4*, *HDAC2*, *GATAD2A* and *KDM1A* followed by RNA-seq analysis. To support the notion that CARM1 and NuRD largely localize on the same regions on chromatin, the impact of knockdown of CHD4, HDAC2, GATAD2A and KDM1A on gene expression was well correlated with that of CARM1 (Supplementary Figure S4A–D). In total, 441 genes were significantly and positively regulated (FC ≥ 1.2) by CARM1 and NuRD in common, of which around 70% had CARM1 binding on their promoters, suggesting that CARM1 largely regulated these genes directly (Figure 4A). In contrast, for genes negatively-regulated by CARM1 and NuRD in common, only around 30% of them exhibited CARM1 and NuRD binding on promoter regions, which was close to background considering the large number of CARM1-bound promoter sites (*n* = 8250) detected in the genome. The impact of knockdown of CARM1, CHD4, HDAC2, GATAD2A and KDM1A on the expression of these 441 genes positively-regulated by CARM1 was shown by heat map and box plot (Figure 4B and C). Importantly, for these CARM1 and NuRD commonly regulated genes, cell cycle was one of the most enriched terms when we performed gene ontology (GO) analysis (Supplementary Figure S4E). Regulation of cell cycle genes by CARM1, CHD4, HDAC2, GATAD2A and KDM1A was validated by RT-qPCR analysis for representative genes (Figure 4D). We then tested whether CARM1 and NuRD regulate cell cycle progression. HEK293 cells were transfected with control siRNA or siRNA specifically targeting *CARM1*, *CHD4*, *HDAC2*, *GATAD2A* or *KDM1A* followed by cell proliferation measurement and FACS analysis. Cell proliferation rate was decreased significantly and number of cells in G1 was increased when knocking down CARM1 or each of these subunits in the NuRD complex (Figure 4E and F).

The transcriptional activation of representative cell cycle genes appeared to be associated with CARM1’s enzymatic activity as wild-type CARM1 (CARM1 (WT)) partially rescued the effects of CARM1 knockdown, but its enzymatically deficient mutant (CARM1 (M)) did so in a much less extent (Figure 4G). Accordingly, regulation of cell cycle progression by CARM1 was dependent on its enzymatic activity (Figure 4H). CARM1 (WT) and (M) were expressed equally well (Figure 4I). Enzymatic activity-dependency to regulate transcription and cell cycle progression by CARM1 was also demonstrated by doing rescue experiments in CARM1-knockout (KO) cells (Supplementary Figure S4F–H). The expression of representative cell cycle genes, cell proliferation, and cell cycle progression were inhibited by EZM2302, further supporting that the regulation of these events by CARM1 is dependent on its enzymatic activity (Figure 4J–L). Taken together, our data revealed that CARM1 and NuRD regulate a large cohort of genes to control cell cycle progression.

### CARM1-mediated GATAD2A methylation is involved in NuRD chromatin binding, transcriptional activation of cell cycle genes, and cell cycle progression

**Figure 5.**
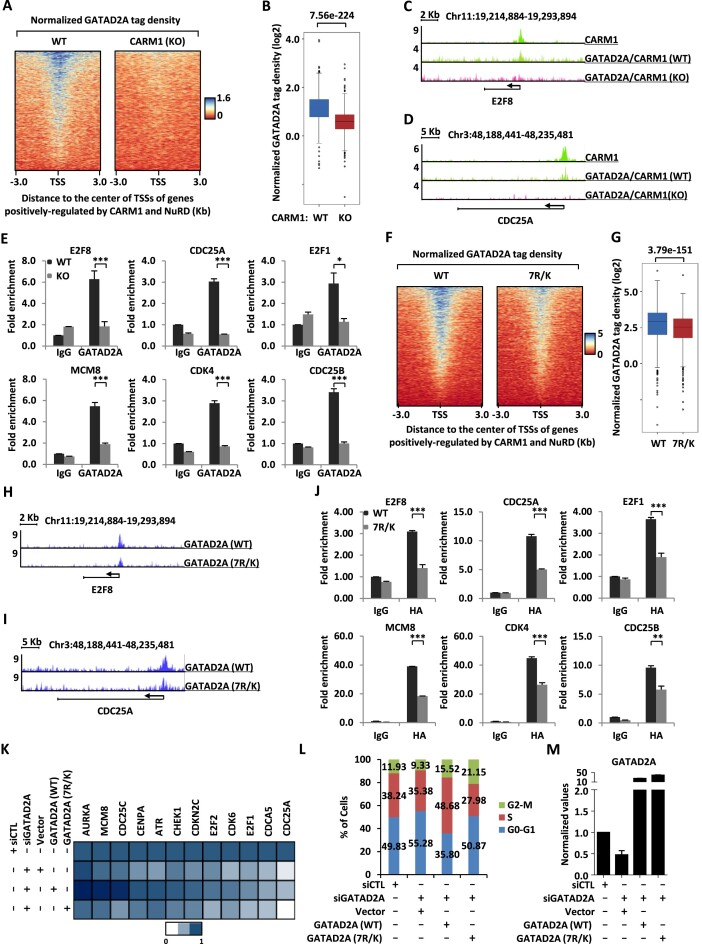
CARM1-mediated hypermethylation of GATAD2A is involved in NuRD chromatin binding, transcriptional activation of cell cycle genes, and cell cycle progression. (**A, B**) GATAD2A ChIP-seq was performed in WT or CARM1 (KO) HEK293 cells, and heat map (A) and box plot (B) representation of GATAD2A ChIP-seq tag density centered on transcription start sites (TSSs) of genes positively-regulated by CARM1 and NuRD is shown. (**C, D**) Genome browser views of CARM1 and GATAD2A ChIP-seq, either in WT or CARM1 (KO) HEK293 cells, on *E2F8* (C) and *CDC25A* (D) gene is shown. (**E**) WT or CARM1 (KO) HEK293 cells were subjected to ChIP with anti-IgG or anti-GATAD2A specific antibody followed by qPCR analysis with primers specifically targeting promoter regions of selected cell cycle genes as indicated (± s.e.m., *P < 0.05, ***P < 0.001). (**F, G**) HEK293 cells were transfected with siRNA targeting 3′UTR region of *GATAD2A* together with or without control vector or vectors expressing HA-tagged WT or mutant GATAD2A (7R/K), followed by ChIP-seq with anti-HA antibody. Heat map (F) and box plot (G) representation of ChIP-seq tag density of WT or mutant GATAD2A (7R/K) HEK293 cells centered on transcription start sites (TSSs) of genes positively-regulated by CARM1 and NuRD. (**H, I**) Genome browser views of GATAD2A WT and GATAD2A mutant (7R/K) ChIP-seq on *E2F8* (H) and *CDC25A* (I) gene are shown. (**J**) HEK293 cells as described in (F) were subjected to ChIP-qPCR analysis with primers specifically targeting the promoter region of selected cell cycle genes as indicated (± s.e.m., **P < 0.01, ***P < 0.001). (**K**) HEK293 cells were transfected with control siRNA or siRNA targeting 3′UTR region of *GATAD2A* together with or without control vector or vectors expressing HA-tagged WT or mutant GATAD2A (7R/K), followed by RT-qPCR analysis to examine the expression of selected cell cycle genes as indicated, and data was represented by heat map. (**L**) HEK293 cells as described in (K) were subjected to FACS analysis. (**M**) HEK293 cells as described in (K) were subjected to RT-qPCR analysis to examine the expression of GATAD2A.

Enzymatic activity-dependency of CARM1 in the transcriptional activation of cell cycle genes and cell cycle progression suggested that CARM1-mediated GATAD2A methylation might be functionally important. Firstly, we tested whether CARM1-mediated methylation is involved in GATAD2A and therefore NuRD binding on chromatin. ChIP-seq analysis of GATAD2A was performed in both WT and CARM1 (KO) HEK293 cells. The results showed that GATAD2A binding was significantly attenuated on genes regulated by CARM1 and NuRD in common in CARM1 KO cells (Figure 5A and B). GATAD2A binding in WT or CARM1 KO cells was shown on representative genes (Figure 5C, D and Supplementary Figure S5A–C). The impact of knockdown of CARM1 on GATAD2A binding was further validated by ChIP-qPCR analysis for representative genes (Figure 5E). CARM1 effects on GATAD2A chromatin binding appeared to be largely dependent on CARM1’s enzymatic activity as CARM1 (WT) largely rescued GATAD2A binding on chromatin, while CARM1 (M) did so in a much less extent (Supplementary Figure S5D). We also examined the binding of other subunits in the NuRD complex, exemplified by HDAC2 and KDM1A, in response to CARM1 depletion, and found that binding of HDAC2 and KDM1A was also significantly reduced (Supplementary Figure S5E–H). Collaborating with the observation that GATAD2A was required for the integrity of NuRD complex, knockdown of GATAD2A led to significantly decreased binding of CARM1 (Supplementary Figure S5I and J). Also, the binding of another subunit of NuRD, CHD4, was significantly decreased when GATAD2A was knocked down (Supplementary Figure S5K). Secondly, ChIP-seq analysis of GATAD2A (WT) and 7R/K mutant was performed, and the binding of 7R/K mutant was found to be significantly lower on genes co-regulated by CARM1 and NuRD compared to GATAD2A (WT) (Figure 5F and G). Binding of GATAD2A (WT) and 7R/K mutant was shown on representative genes (Figure 5H, I and Supplementary Figure S5L–N), and further validated by ChIP-qPCR analysis (Figure 5J).

We next examined whether CARM1-mediated methylation on GATAD2A is involved in the transcriptional activation of CARM1 and NuRD commonly regulated genes and cell cycle progression. HEK293 cells were transfected with control siRNA or siRNA specifically targeting *GATAD2A* in the presence or absence of control vector or vector expressing GATAD2A (WT) or 7R/K mutant, followed by gene expression and cell cycle analysis. It was found that, compared to GATAD2A (WT), the 7R/K mutant was much less efficient to rescue the gene expression defect and cell cycle arrest caused by GATAD2A knockdown (Figure 5K and L). GATAD2A (WT) and 7R/K mutant expressed equally well as examined by both RT-qPCR and immunoblotting analysis (Figure 5M and Supplementary Figure S5O). It should be noted that the 7R/K mutant appeared to have no impact on the integrity of the NuRD complex compared to GATAD2A (WT), which was consistent with our observation that CARM1 exhibited no significant impact on the integrity of the NuRD complex (Supplementary Figure S5P). Taken together, our data suggested that CARM1-mediated methylation is involved in the chromatin binding of the NuRD complex, the transcriptional activation of cell cycle genes, and cell cycle progression.

### CARM1-mediated GATAD2A methylation is required for breast cancer cell growth both *in vitro* and *in vivo*

**Figure 6.**
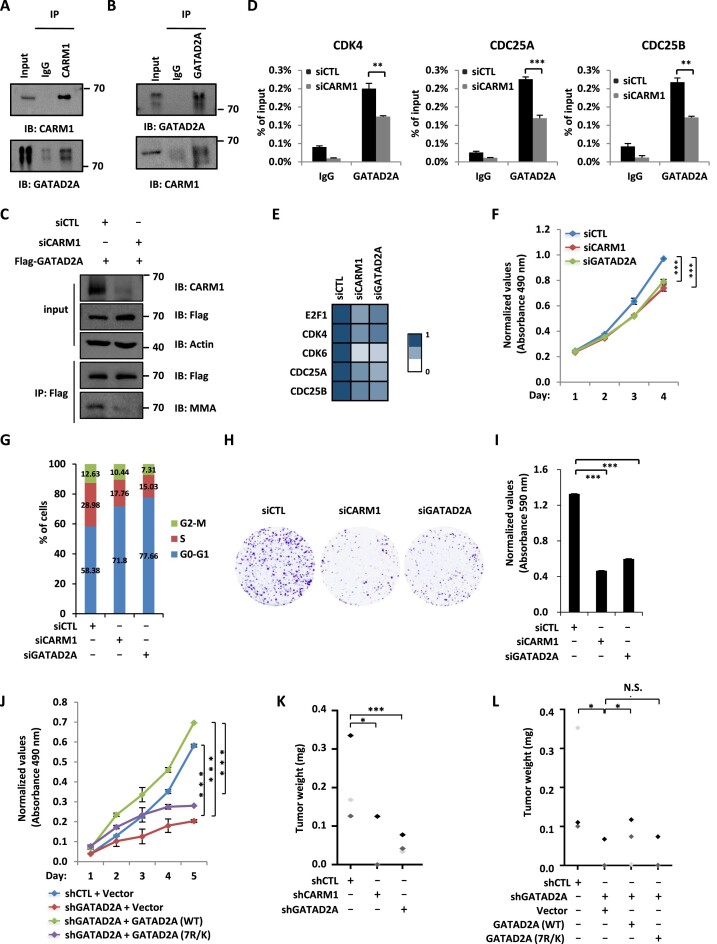
CARM1-mediated GATAD2A methylation is required for breast cancer cell growth both *in vitro* and *in vivo*. (**A, B**) MCF7 cells were subjected to co-immunoprecipitation (Co-IP) assay with control IgG, anti-GATAD2A (A), or anti-CARM1 (B) antibody and followed by immunoblotting (IB) with antibodies as indicated. (**C**) MCF7 cells were transfected with siRNA (*siCTL*) or siRNA specific against *CARM1* (si*CARM1*) and then infected with lenti-viral vector expressing Flag-tagged GATAD2A, followed by immunoprecipitation (IP) with anti-Flag antibody and immunoblotting (IB) analysis with antibodies as indicated. (**D**) MCF7 cells transfected with siRNA (*siCTL*) or siRNA specific against *CARM1* (si*CARM1*) were subjected to ChIP with control IgG or anti-GATAD2A specific antibody, followed by qPCR analysis with primers specifically targeting promoter regions of selected cell cycle genes as indicated (± s.e.m., ***P < 0.001). (**E-H**) MCF7 cells transfected with control siRNA (*siCTL*) or siRNA specific against *CARM1* (si*CARM1*) or *GATAD2A* (si*GATAD2A*) were subjected to RNA extraction and RT-qPCR analysis to examine the expression of selected cell cycle genes as indicated (E), cell proliferation assay (F), FACS analysis (G), and colony formation assay (H) (± s.e.m., ***P < 0.001). (**I**) Quantification of the crystal violet dye as shown in (H). (**J**) MCF7 cells were infected with control shRNA (shCTL) or shRNA targeting 3′UTR region of GATAD2A together with or without control lentiviral vector or vectors expressing WT or mutant GATAD2A (7R/K), followed by cell proliferation assay (± s.e.m., ***P < 0.001). (**K**) MCF7 cells infected with control shRNA (shCTL) or shRNA specific against CARM1 or GATAD2A, were injected subcutaneously into female BALB/C nude mice and brushed with estrogen (E_2_, 10^−2^ M) on the neck every two days for six weeks. Mice were then euthanized and tumors were excised and weighted (± s.e.m., *P < 0.05, ***P < 0.001). (**L**) MCF7 cells as described in (E) were injected subcutaneously into female BALB/C nude mice and brushed with estrogen (E_2_, 10^−2^ M) on the neck every two days for six weeks. Mice were then euthanized and tumors were excised and weighted (± s.e.m., *P < 0.05, N.S., not significant).

The functional role of CARM1-mediated GATAD2A methylation in cell cycle gene transcriptional activation and cell cycle progression prompted us to examine its role in cancer. We focused on breast cancer in the current study as numerous studies including ours showed that CARM1 plays a critical role in breast cancer development (5,68). Both CARM1 and GATAD2A were found to be expressed higher in breast tumor than normal samples, and their high expression predict poor prognosis in breast cancer patients (Supplementary Figure S6A–D). The direct interaction between CARM1 and GATAD2A was verified in MCF7 cells with co-immunoprecipitation analysis (Figure 6A and B). We then performed ChIP-seq for both CARM1 and GATAD2A in MCF7 cells. Tag density plot and heat map demonstrated that both proteins were largely co-localized in the genome (Supplementary Figure S6E and F). ChIP-seq tag density of CARM1 was highly correlated to that of GATAD2A (Supplementary Figure S6G and H). Binding of CARM1and GATAD2A detected from ChIP-seq on representative genes was shown (Supplementary Figure S6I–K). Knockdown of CARM1 led to a significant reduction of GATAD2A methylation as well as GATAD2A binding on the promoter regions of representative cell cycle genes (Figure 6C and D). Consequently, knockdown of CARM1 and GATAD2A resulted in a decreased expression of these cell cycle genes (Figure 6E).

Next, we examined whether CARM1-madiated GATAD2A methylation is required for breast cancer cell growth and tumorigenesis. Cell proliferation rate was decreased significantly and cells were arrested at G1 phase when CARM1 or GATAD2A was knocked down in MCF7 cells (Figure 6F and G). The effects on MCF7 cell growth were further demonstrated by colony formation assay (Figure 6H and I). Knockdown efficiency of siCARM1 and siGATAD2A was demonstrated by RT-qPCR analysis (Supplementary Figure S6L). The effects of CARM1 and GATAD2A on cell proliferation, cell cycle progression, and colony formation were also demonstrated in MDA-MB-231 cells (Supplementary Figure S6M–P). We then tested whether CARM1-mediated GATAD2A methylation is involved in cell proliferation of MCF7 cells by transfecting cells with control shRNA or shRNA specifically targeting *GATAD2A* in the presence or absence of control vector or vector expressing GATAD2A (WT) or 7R/K mutant, followed by cell proliferation analysis. The 7R/K mutant was less efficient to rescue the defects in cell proliferation caused by GATAD2A knockdown (Figure 6J). We further tested the effect of CARM1 and GATAD2A on tumor growth *in vivo* by injecting nude mice subcutaneously with control MCF7 cells or cells infected with shRNA targeting *CARM1* or *GATAD2A*, and then treated with estrogen to stimulate tumor growth. Knockdown of CARM1 and GATAD2A significantly attenuated estrogen-induced tumorigenesis (Figure 6K). Knockdown efficiency of shCARM1 and shGATAD2A was demonstrated by RT-qPCR analysis (Supplementary Figure S6Q). The involvement of CARM1-mediated GATAD2A methylation in tumor growth was also tested. In consistent with what we observed in cultured cells, GATAD2A 7R/K mutant was less effective in rescuing the tumor growth defects caused by GATAD2A knockdown (Figure 6L). GATAD2A (WT) and 7R/K mutant were expressed equally well (Supplementary Figure S6R). Taken together, our data suggested that CARM1-mediated GATAD2A methylation is involved in breast cancer cell growth and tumorigenesis.

### Targeting CARM1 with CARM1 inhibitors inhibits breast cancer cell growth both *in vitro* and *in vivo*

**Figure 7.**
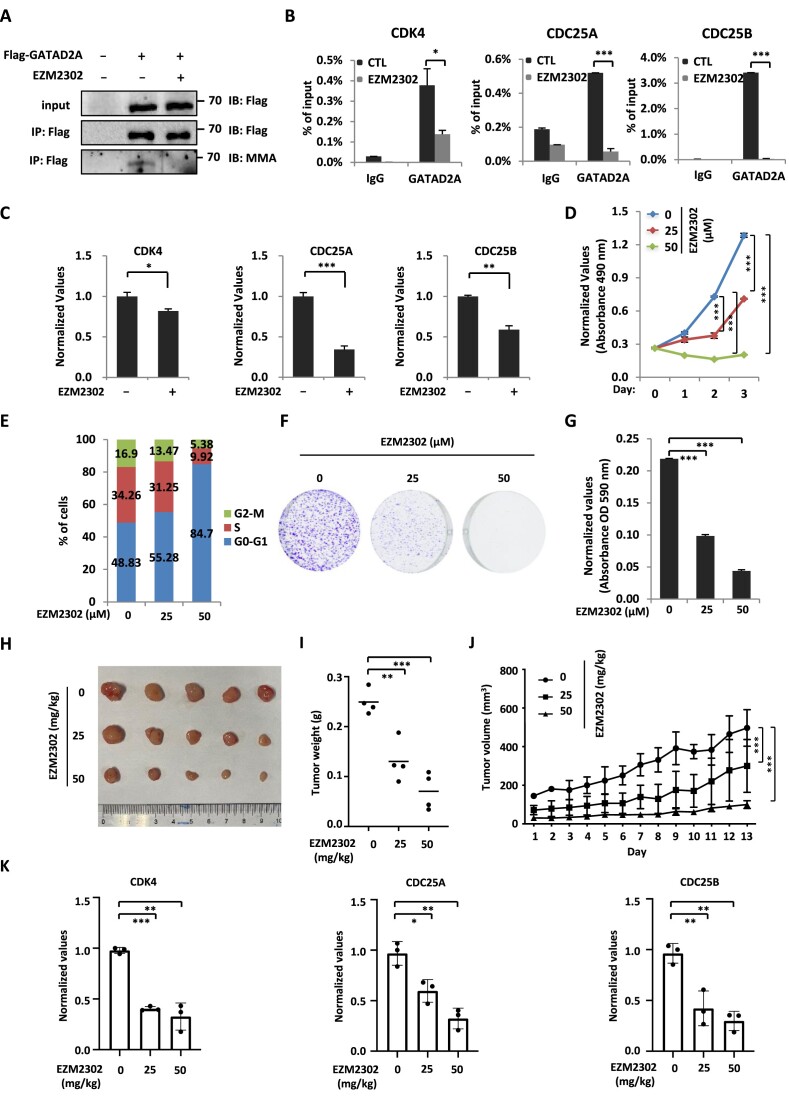
Targeting CARM1 with EZM2302 inhibits breast cancer cell growth both *in vitro* and *in vivo*. (**A**) MCF7 cells were transfected with or without Flag-tagged GATAD2A and treated with or without EZM2302 (25 μM, 48 h), followed by immunoprecipitation (IP) with anti-Flag antibody and immunoblotting (IB) analysis with antibodies as indicated. (**B**) MCF7 cells treated with or without EZM2302 (25 μM, 48 h) were subjected to ChIP with control IgG or anti-GATAD2A specific antibody followed by qPCR analysis with primers specifically targeting promoter regions of selected cell cycle genes as indicated (± s.e.m., *P < 0.05, ***P < 0.001). (**C**) MCF7 cells treated with or without EZM2302 (25 μM, 48 h) were subjected to RNA extraction and RT-qPCR analysis to examine the expression of selected cell cycle genes as indicated (± s.e.m., *P < 0.05, **P < 0.01, ***P < 0.001). (**D-F**) MCF7 cells treated with or without EZM2302 at concentration as indicated were subjected to cell proliferation assay (D), FACS analysis (E), and colony formation assay (F) (± s.e.m., **P < 0.01, ***P < 0.001). (**G**) Quantification of the crystal violet dye as shown in (F) (± s.e.m., ***P < 0.001). (**H**) MCF7 cells were injected subcutaneously into female BALB/C nude mice, and brushed with estrogen (E_2_, 10^−2^ M) on the neck every two days until tumor size reached approximately 100 mm^3^. Mice were then randomly assigned into three groups and treated with or without EZM2302 intraperitoneally every two days for 13 days. Tumors were harvested, photographed, and weighted. (**I**) The weight of tumors in (H) is shown (± s.e.m., **P < 0.01, ***P < 0.001). (**J**) The growth curve of tumors in (H) is shown. (**K**) Tumors as described in (H) were subjected to RNA extraction and RT-qPCR analysis to examine the expression of selected cell cycle genes as indicated (± s.e.m., *P < 0.05, **P < 0.01, ***P < 0.001).

The functional role of CARM1-mediated GATAD2A methylation in breast cancer cell growth prompted us to examine whether targeting CARM1-mediated GATAD2A methylation using CARM1 inhibitors, EZM2302 and TP-064, could inhibit breast cancer cell growth. MCF7 cells infected with lentivirus expressing GATAD2A were treated with EZM2302 followed by immunoprecipitation and immunoblotting to detect GATAD2A methylation. EZM2302 treatment led to a significant reduction of GATAD2A methylation (Figure 7A). Consequently, GATAD2A binding on the promoter regions on representative cell cycle genes and the expression of these cell cycle genes were significantly attenuated in the presence of EZM2302 (Figure 7B and C). Furthermore, cell proliferation rate was decreased significantly and cells were arrested at G1 phase when MCF7 cells were treated with EZM2302 (Figure 7D and E). The effects of EZM2302 on MCF7 cell growth were also demonstrated by colony formation assay (Figure 7F and G). Another inhibitor of CARM1, TP-064, was also found to be effective in inhibiting MCF7 cell proliferation, cell cycle progression, and colony formation (Supplementary Figure S7A–D) (69). Furthermore, EZM2302 significantly attenuated the weight and growth rate of MCF7 cells-derived tumors in mice (Figure 7H–J), while it displayed minimal effects on the body weight of mice (Supplementary Figure S7E). To link CARM1-regulated cell cycle gene transcription to EZM2302 effects on tumor growth, the expression of cell cycle genes including CDK4, CDC25A and CDC25B was significantly inhibited by EZM2302 treatment (Figure 7K).

To further strengthen the functional role of CARM1-mediated GATAD2A methylation in breast cancer, EZM2302 significantly inhibited GATAD2A methylation and GATAD2A binding on the promoter regions of representative cell cycle genes in MDA-MB-231 cells (Supplementary Figure S7F and G). The expression of these genes was reduced with EZM2302 treatment (Supplementary Figure S7H). Consequently, cell proliferation, cell cycle progression, and colony formation were significantly inhibited when MDA-MB-231 cells were treated with EZM2302 (Supplementary Figure S7I–L). Furthermore, EZM2302 significantly inhibited the weight and growth of MDA-MB-231 cells-derived tumors, while exhibited no significant impact on body weight (Supplementary Figure S7M–P). The expression of representative cell cycle genes was found to be significantly inhibited by EZM2302 in tumor samples (Supplementary Figure S7Q). Taken together, our data suggested that targeting CARM1 is effective in inhibiting breast cancer cell growth both *in vitro* and *in vivo*.

## Discussion

**Figure 8.**
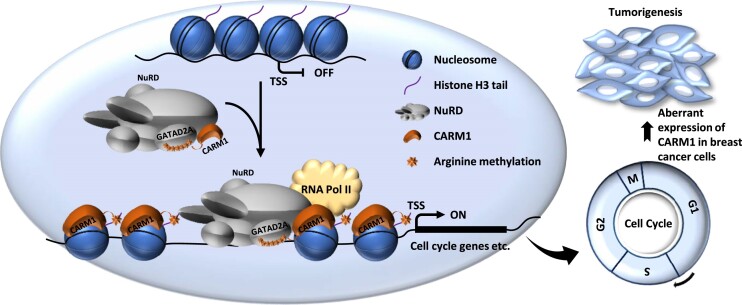
A proposed model of CARM1-mediated NuRD methylation in gene transcriptional regulation and cell cycle control. CARM1 and NuRD commonly bind and activate a large cohort of genes with implications in cell cycle control to facilitate G1/S transition. Activation of this gene program requires CARM1 to methylate a key subunit in NuRD, GATAD2A/2B. Aberrant expression of CARM1 and NuRD results in uncontrolled expression of these cell cycle genes and cell cycle progression, leading to cancers, such as breast cancer.

CARM1 was identified to contain histone arginine methyltransferase activity and serves as a transcriptional coactivator. However, the substrates utilized by CARM1 to orchestrate transcriptional regulation remain incompletely understood. In the current study, we found that CARM1 and NuRD interact, occupy a large number of chromatin sites in common, commonly regulate the transcriptional activation of a set of cell cycle genes, and promote cell cycle progression. CARM1 methylates one of the subunits, GATAD2A, in NuRD to regulate its binding with chromatin and function in gene transcription and cell cycle control. The clinical relevance of CARM1-mediated GATAD2A methylation in cell cycle control is further illustrated in breast cancer, where higher expression of CARM1 and GATAD2A is observed compared to normal tissues. Knockdown of CARM1 and GATAD2A significantly attenuates breast cancer cell growth both *in vitro* and *in vivo*. Targeting CARM1 with EZM2302, a CARM1-specific inhibitor, significantly inhibits CARM1-mediated GATAD2A methylation, cell cycle gene transcription, cell cycle progression, and breast tumor growth (Figure 8).

Despite that CARM1 has been shown to interact with a plethora of proteins and have a large set of substrates, a deep characterization of its interactome is still needed to fully understand its diverse functions in both physiological and pathological conditions. Through affinity purification and mass spectrometry analysis, we demonstrated that, in addition to the SWI/SNF, CARM1 also interacts with another chromatin remodeling complex, the NuRD complex. The observed interaction between CARM1 and NuRD is intriguing to us for several reasons. Firstly, despite that NuRD was initially thought to exclusively repress gene transcription, genome-wide mapping experiments suggested that NuRD, including CHD3/Mi-2a, CHD4/Mi-2b, GATAD2A and GATAD2B sub-complexes, might be tightly linked to transcriptional activation. The observed interaction between CARM1 and NuRD suggests that CARM1 might provide a molecular basis for NuRD in gene activation. Secondly, both CARM1 and NuRD have been shown to control cell cycle progression, but whether they cooperate to do so and the underlying molecular mechanisms remain elusive. Finally, during the course of mapping CARM1 substrates in the proteome, the methylation of GATAD2A/2B subunit in the NuRD complex was found to be dependent on CARM1, suggesting a functional connection between CARM1 and the NuRD complex (6). It should also be noted that, though CHD3/Mi-2a and CHD4/Mi-2b or GATAD2A and GATAD2B sub-complexes were shown to be mutually exclusive, both were found to interact with CARM1, suggesting that CARM1 might be a common molecule utilized by all sub-complexes to regulate gene expression. CARM1 specifically and directly interacts with GATAD2A/2B through β-strands in the N-terminus (aa 63–88), which appeared to be different from the interacting region that CARM1 utilizes to target other substrates, such as MED12 and histone H3. Interestingly, several other complexes were also identified to be associated with CARM1 including the DNA replication factor C complex, the THO complex, the Prp19 complex, the TFIIIC complex, among others, suggesting CARM1 might also play important roles in cellular processes such as DNA replication, RNA splicing, and tRNA transcription.

The connection between CARM1 and NuRD was further demonstrated on chromatin. The co-localization of CARM1 and NuRD on chromatin prompted us to examine whether they regulate gene transcription in a similar fashion. Knockdown of CARM1 or NuRD resulted in a moderate but significant and reproducible effect on a large cohort of genes. Among all the functions related to the genes regulated by CARM1 and NuRD, cell cycle regulation was one of the most prevalent. Indeed, the cumulative effects of CARM1 and NuRD co-regulated transcription were essential for cell cycle progression. The NuRD complex was shown to be recruited to and function locally on DNA damage sites (50,70). In our transcriptomics analysis herein, we noted that a cohort of p53 target genes with implications in DNA damage response were also regulated by CARM1 and NuRD, suggesting they might function in DNA damage response through regulating gene transcription, which adds another layer of complexity to the means of NuRD function in DNA damage response. CARM1 and NuRD complex's function in gene transcriptional regulation in response to DNA damage signals as well as the connection with p53 protein is worthy of future investigation.

In addition of methylating histones to activate gene transcription, CARM1 also methylated the GATAD2A subunit in the NuRD complex to control its recruitment onto chromatin. A unique feature of CARM1-mediated arginine methylation was that the arginine residues being methylated form a cluster, exhibiting a hypermethylation status, such that there were seven arginine residues in total found to be methylated in a window <100 amino acids in GATAD2A. This type of hypermethylation appeared to be a common strategy utilized by CARM1 to target its substrates, such as MED12 and Notch1 as previously reported (9,20), and many others identified in our experiments to globally map CARM1 substrates (5,6). Notably, H4R3me2a, H3R17me2a and methylated arginine 1810 (R1810) in the C-terminal domain (CTD) of RNA polymerase II (RNA Pol II) mediated by CARM1 were shown to be recognized by the tudor-domain of TDRD3, which functions as a transcriptional coactivator (21,71). Therefore, we propose that GATAD2A methylation might recruit arginine methylation readers such as TDRD3 to activate genes involved in cell cycle regulation. We propose that this type of hypermethylation might serve as ‘amplifier’ to ensure the recruitment of methylation reader proteins to activate gene transcription.

Due to the fact that they are highly expressed in breast tumor samples and their high expression predict poor prognosis in patients with breast cancer, the pathological relevance of CARM1 and NuRD was investigated. We demonstrated that CARM1-mediated GATAD2A methylation was important for the growth of breast cancer cells both *in vitro* and *in vivo*. CARM1’s function in breast cancer is, at least partially, mediated through its methyltransferase activity targeting GATAD2A. Our data thereby suggested that CARM1-mediated GATAD2A methylation might serve as a potential druggable target in breast cancer. Indeed, EZM2302, a CARM1-specific inhibitor, inhibits CARM1-mediated GATAD2A methylation, GATAD2A chromatin binding, transcriptional activation of cell cycle genes, cell cycle progression, and eventually tumor growth. Therefore, targeting CARM1 enzymatic activity or peptide mimics interfering CARM1-mediated GATAD2A/2B methylation will provide a new therapeutic avenue for treating breast cancer as well as other CARM1-dependent cancers.

## Supplementary Material

gkae329_Supplemental_Files

## Data Availability

The authors confirm that the data supporting the findings of this study are available within the article and/or its supplementary data, or can be made available upon reasonable request. ChIP-seq and RNA-seq files were deposited in the Gene Expression Omnibus database under accession GSE209910.
