# Supplementary material for: CARM1 hypermethylates the NuRD chromatin remodeling complex to promote cell cycle gene expression and breast cancer development
Source: Nucleic Acids Res. 2024 Apr 27;52(12):6811–29. doi: 10.1093/nar/gkae329 (PMC11229315; doi:10.1093/nar/gkae329)
Supplement: gkae329_Supplemental_Files [file gkae329_supplemental_files.zip › Supplementary information.pdf]

Supplementary Information

Supplementary Figure Legends

Figure S1. CARM1 interacts with the NuRD complex.

(A) HEK293 cells stably expressing Flag-HA-tagged CARM1 were subjected to immunoblotting (IB) with antibodies as indicated.

(B) Subunits in the SWI/SNF complex, both BAF and PBAF sub-complexes, identified from mass spectrometry analysis as described in Fig. 1A and the corresponding number of unique peptides are shown as indicated.

(C)The overlapping of proteins that were associated with and methylated by CARM1 is shown by venn diagram.

(D) Immunoprecipitates as described in Fig. 1C were subjected to immunoblotting (IB) with antibodies as indicated.

(E) HEK293 cells as described in Fig. 1A were subjected to immunoprecipitation (IP) with anti-HA antibody followed by immunoblotting (IB) with antibodies as indicated.

(F) GST pull-down assay was performed by mixing Flag-tagged, individual subunit in the NuRD complex purified from HEK293 cells with GST or GST-tagged CARM1 followed by immunoblotting (IB) with anti-Flag antibody as described in Fig. 1E.

(G) The expression of bacterially-expressed, GST-tagged CARM1 was detected by coomassie blue staining (C.B.S) (left panel) or immunoblotting (IB) (right panel).

GST and GST-CARM1 proteins were indicated by arrowhead and bracket, respectively.

(H) HEK293 cells transfected with control siRNA (siCTL) or siRNA specifically targeting GATAD2A (siGATAD2A) were subjected to immunoprecipitation (IP) with anti-CHD4 antibody followed by mass spectrometry analysis. The list of subunits in the NuRD complex identified, the number of peptides for each subunit, and the ratio of the abundance of each subunit in WT and GATAD2A (KD) cells are shown.

(I) Schematic representation of CARM1 N-terminal truncations.

(J, K) *In vitro* GST pull-down assay was performed by mixing purified, Flag-tagged GATAD2A with GST or GST-tagged CARM1 truncations as described in (I) followed by immunoblotting (IB) using anti-Flag antibody. The expression of CARM1 truncations was examined by coomassie blue staining (C.B.S) and indicated by black arrowheads.

(L) HEK293 cells were transfected with vectors expressing HA-tagged MED12 C-terminus (1616–2177) and empty vector, full length (FL), or region 3-deleted (Δ3) CARM1 followed by immunoprecipitation (IP) with anti-HA antibody and immunoblotting (IB) with anti-Flag or anti-HA antibody.

Figure S2. CARM1 hypermethylates GATAD2A/2B in the NuRD complex.

(A, B) Global mapping of CARM1 substrates by high-resolution mass spectrometry (MS) analysis of enriched peptides from anti-monomethyl- and anti-asymmetric

dimethyl-arginine antibodies in SILAC (stable isotope labeling by amino acids) labeled wild-type (WT) and CARM1 knockout (KO) HEK293 cells was performed, and the arginine methylation sites recovered in GATAD2A (A) and GATAD2B (B) in the NuRD complex and the corresponding abundance in WT and CARM1 KO cells are shown. me1: mono-methylation; me2: di-methylation.

(C, D) *In vitro* methylation assay was performed by mixing core histones (C) or MED12 (1,616-2,177) (D) with full length (FL), region 3-deleted (Δ3), amino (N)-terminal-deleted (ΔN) or enzymatic dead (M) CARM1, followed by immunoblotting (IB) with antibodies as indicated.

(E) Wild-type (WT) and CARM1 knockout (KO) HEK293 cell lines generated by CRISPR/Cas9 system were subjected to immunoblotting (IB) with antibodies as indicated

(F) Amino acid sequence of human GATAD2A. Arginine residues identified to be with only mono-methylation were highlighted in red, and those with both mono- and di-methylation were highlighted in red and underlined.

(G) *In vitro* methylation assay as described in Fig. 2H was subjected to autoradiogram.

Figure S3. CARM1 and NuRD complex occupy a large number of chromatin sites in common.

(A-D) Genome-wide correlation between the ChIP-seq tag density of CARM1 and CHD4 (A), HDAC2 (B), GATAD2A (C) or KDM1A (D).

(E-H) Genome browser views of CARM1, CHD4, HDAC2, GATAD2A and KDM1A ChIP-seq on *CDK6* (E), *E2F1* (F), *CDC20* (G) and *CDK4* (H) gene regions.

Figure S4. CARM1 and NuRD activate a large set of cell cycle genes to promote cell cycle progression in a CARM1 enzymatic activity-dependent manner.

(A-D) The Correlation between the effects of CARM1 and subunits in the NuRD complex including CHD4 (A), HDAC2 (B), GATAD2A (C), and KDM1A (D) on whole transcriptome based on RNA-seq.

(E) Gene ontology (GO) analysis of genes positively-regulated by CARM1 and NuRD as shown in Fig. 4A.

(F-H) Wild-type (WT) and CARM1 knockout (KO) HEK293 cells were transfected with control vector or vector expressing wild-type CARM1 (WT) or its enzymatically dead mutant (M), followed by RT-qPCR analysis to examine the expression of selected cell cycle genes (F) and CARM1 (H) as indicated, and FACS analysis to check cell cycle progression (G).

Figure S5. CARM1-mediated hypermethylation of GATAD2A is involved in NuRD chromatin binding, transcriptional activation of cell cycle genes, and cell cycle progression.

(A-C) Genome browser views of ChIP-seq of CARM1 and GATAD2A, either in WT or CARM1 (KO) HEK293 cells, on *E2F1* (A), *CDC20* (B) and *CDK4* (C) gene regions are shown.

(D) Wild-type (WT) and CARM1 knockout (KO) HEK293 cells were transfected with control vector or vector expressing wild-type CARM1 (WT) or its enzymatically deficient mutant (M), followed by ChIP with anti-GATAD2A antibody and qPCR analysis with primers specifically targeting promoter regions of selected cell cycle genes as indicated (± s.e.m., ***$p < 0.001$).

(E, F) Heat map (E) and box plot (F) representation of HDAC2 ChIP-seq tag density in WT or CARM1 (KO) HEK293 cells centered on transcription start sites (TSSs) of genes positively-regulated by CARM1 and NuRD.

(G, H) Heat map (G) and box plot (H) representation of KDM1A ChIP-seq tag density in WT or CARM1 (KO) HEK293 cells centered on transcription start sites (TSSs) of genes positively-regulated by CARM1 and NuRD.

(I, J) HEK293 cells transfected with siRNA (*siCTL*) or siRNA specific against *GATAD2A* (*siGATAD2A*) were subjected to CARM1 ChIP-seq analysis. Heat map (J) and box plot (K) representation of CARM1 ChIP-seq tag density centered on CARM1 binding sites is shown.

(K) HEK293 cells transfected with siRNA (*siCTL*) or siRNA specific against *GATAD2A* (*siGATAD2A*) were subjected to ChIP with control IgG or anti-CHD4 specific antibody, followed by qPCR analysis with primers specifically targeting

promoter regions of selected cell cycle genes as indicated (± s.e.m., *p < 0.05, **p < 0.01, ***p < 0.001).

(L-N) Genome browser views of GATAD2A (WT) and GATAD2A mutant (7R/K) ChIP-seq on *E2F1* (I), *CDC20* (J) and *CDK4* (K) gene regions are shown.

(O) HEK293 cells as described in Fig. 5K were subjected to immunoblotting (IB) analysis using antibodies as indicated.

(P) HEK293 cells transfected with siRNA targeting 3'UTR region of *GATAD2A* in the presence of vector expressing Flag-tagged GATAD2A (WT) or 7R/K mutant were subjected to immunoprecipitation (IP) with anti-Flag antibody followed by immunoblotting (IB) analysis using antibodies as indicated.

Figure S6. CARM1-mediated GATAD2A methylation is required for breast cancer cell growth both *in vitro* and *in vivo*.

(A, B) Box plot showing the expression of CARM1 (A) and GATAD2A (B) (FPKM) in a cohort of clinical breast cancer (n = 1,102) and normal (n = 113) samples from TCGA (The Cancer Genome Atlas).

(C, D) Kaplan-Meier survival analyses for OS (overall survival) (n = 1,402) of breast cancer patients using CARM1 (C) or GATAD2A (D) as input.

(E, F) Histogram (E) and heat map (F) representation of CARM1 and GATAD2A ChIP-seq tag density centered on CARM1 binding sites. bp: base pair.

(G) Box plot representation of ChIP tag density (log2) of GATAD2A on CARM1 binding sites, which were divided into three sub-classes, high, medium and low based on ChIP-seq tag density (± s.e.m., ***p < 0.001).

(H) Genome-wide correlation between the ChIP-seq tag density of CARM1 and GATAD2A.

(I-K) Genome browser views of CARM1 and GATAD2A ChIP-seq on CDK4 (I), CDC25A (J), and CDC25B (K) gene regions.

(L) The knockdown efficiency of si*CARM1* and si*GATAD2A* as described in Fig. 6E-6I was examined by RT-qPCR analysis.

(M-O) MDA-MB-231-231 cells transfected with control siRNA (*siCTL*) or siRNA specific against *CARM1* (si*CARM1*) or *GATAD2A* (si*GATAD2A*) were subjected to cell proliferation assay (M), FACS analysis (N), and colony formation assay (O) (± s.e.m., **p < 0.01, ***p < 0.001).

(P) Quantification of the crystal violet as shown in (O) (± s.e.m., ***p < 0.001).

(Q) The knockdown efficiency of sh*CARM1* and sh*GATAD2A* as described in Fig. 6K was examined by RT-qPCR analysis.

(R) Cells as described in Fig. 6J and 6L were subjected to immunoblotting (IB) analysis using antibodies as indicated.

Figure S7. Targeting CARM1 with EZM2302 inhibits breast cancer cell growth both *in vitro* and *in vivo*.

(A-C) MCF7 cells treated with or without TP-064 at concentration as indicated were subjected to cell proliferation assay (A), FACS analysis (B), and colony formation assay (C) (± s.e.m., *$p < 0.05$, ***$p < 0.001$).

(D) Quantification of the crystal violet dye as shown in (C) (± s.e.m., ***$p < 0.001$).

(E) The body weight of mice as described in Fig. 7H is shown.

(F) MDA-MB-231 cells were transfected with or without Flag-tagged GATAD2A and treated with or without EZM2302 (25 μM, 48 h), followed by immunoprecipitation (IP) with anti-Flag antibody and immunoblotting (IB) analysis with antibodies as indicated.

(G) MDA-MB-231 cells treated with or without EZM2302 (25 μM, 48 h) were subjected to ChIP with control IgG or anti-GATAD2A specific antibody followed by qPCR analysis with primers specifically targeting promoter regions of selected cell cycle genes as indicated (± s.e.m.).

(H) MDA-MB-231cells treated with or without EZM2302 (25 μM, 48 h) were subjected to RNA extraction and RT-qPCR analysis to examine the expression of selected cell cycle genes as indicated (± s.e.m., *$p < 0.05$, **$p < 0.01$).

(I-K) MDA-MB-231cells treated with or without EZM2302 at concentration as indicated were subjected to cell proliferation assay (I), FACS analysis (J), and colony formation assay (K) (± s.e.m., **$p < 0.01$, ***$p < 0.001$).

(L) Quantification of the crystal violet dye as shown in (K) (± s.e.m., ***$p < 0.001$).

(M) MDA-MB-231cells were injected subcutaneously into female BALB/C nude mice. Mice were then randomly assigned into three groups when tumor size reached approximately 100 mm^3, and then treated with or without EZM2302 intraperitoneally every two days for 11 days. Tumors were harvested, photographed, and weighted.

(N) The weight of tumors in (M) is shown (± s.e.m., ***P < 0.001).

(O) The growth curve of tumors in (M) is shown.

(P) The body weight of mice as described in (M) is shown.

(Q) Tumors as described in (H) were subjected to RNA extraction and RT-qPCR analysis to examine the expression of selected cell cycle genes as indicated (± s.e.m., *p < 0.05, **p < 0.01).

Supplementary Table Legend

Table S1. MS2 spectrum of arginine-methylated peptides identified in GATAD2A. MS2 spectrum of methylated arginine peptides in GATAD2A as described in Fig. 2E are shown as indicated.

Table S2. Sequence information for all qPCR primers used in the current study. Sequence information of qPCR primers designed to detect gene expression (mRNA) or factor binding on promoter region (ChIP) are shown. F: forward; R: reverse.

Table S3. Antibodies used in the current study. Vendor, catalog number and applications of antibodies used in this study are shown. IB: immunoblotting; IP:

immunoprecipitation; ChIP-seq: chromatin immunoprecipitation coupled with high throughput sequencing.

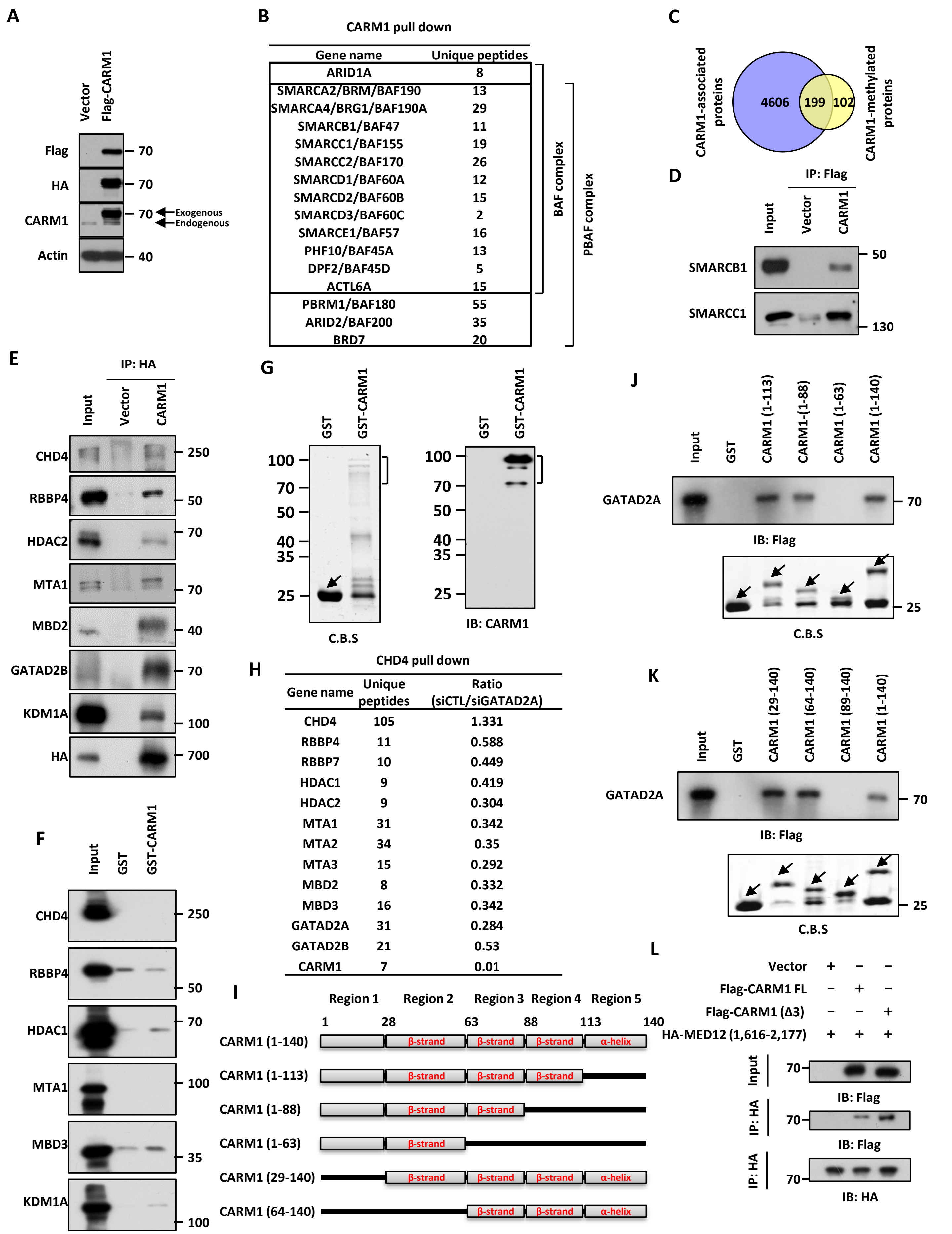
A
Vector
Flag-CARM1
Flag
HA
CARM1
Actin
70
70
70
40
Exogenous
Endogenous
B
CARM1 pull down
Gene name Unique peptides
ARID1A 8
SMARCA2/BRM/BAF190 13
SMARCA4/BRG1/BAF190A 29
SMARCB1/BAF47 11
SMARCC1/BAF155 19
SMARCC2/BAF170 26
SMARCD1/BAF60A 12
SMARCD2/BAF60B 15
SMARCD3/BAF60C 2
SMARCE1/BAF57 16
PHF10/BAF45A 13
DPF2/BAF45D 5
ACTL6A 15
PBRM1/BAF180 55
ARID2/BAF200 35
BRD7 20
BAF complex
PBAF complex
C
CARM1-associated proteins
CARM1-methylated proteins
4606
199
102
D
IP: Flag
Input
Vector
CARM1
SMARCB1
SMARCC1
50
130
E
IP: HA
Input
Vector
CARM1
CHD4
RBBP4
HDAC2
MTA1
MBD2
GATAD2B
KDM1A
HA
250
50
70
70
40
70
100
700
F
Input
GST
GST-CARM1
CHD4
RBBP4
HDAC1
MTA1
MBD3
KDM1A
250
50
70
100
35
100
IB: Flag
G
GST
GST-CARM1
100
70
50
40
35
25
C.B.S
IB: CARM1
H
CHD4 pull down
Gene name Unique peptides Ratio (siCTL/siGATAD2A)
CHD4 105 1.331
RBBP4 11 0.588
RBBP7 10 0.449
HDAC1 9 0.419
HDAC2 9 0.304
MTA1 31 0.342
MTA2 34 0.35
MTA3 15 0.292
MBD2 8 0.332
MBD3 16 0.342
GATAD2A 31 0.284
GATAD2B 21 0.53
CARM1 7 0.01
I
Region 1
Region 2
Region 3
Region 4
Region 5
1
28
63
88
113
140
CARM1 (1-140)
CARM1 (1-113)
CARM1 (1-88)
CARM1 (1-63)
CARM1 (29-140)
CARM1 (64-140)
CARM1 (89-140)
β-strand
α-helix
J
Input
GST
CARM1 (1-113)
CARM1-(1-88)
CARM1 (1-63)
CARM1 (1-140)
GATAD2A
70
IB: Flag
25
C.B.S
K
Input
GST
CARM1 (29-140)
CARM1 (64-140)
CARM1 (89-140)
CARM1 (1-140)
GATAD2A
70
IB: Flag
25
C.B.S
L
Vector + − −
Flag-CARM1 FL − + −
Flag-CARM1 (Δ3) − − +
HA-MED12 (1,616-2,177) + + +
Input
IP: HA
IP: HA
70
IB: Flag
IB: Flag
IB: HA

Figure S1

A

Peptides with arginine methylation	Type of methylation	Abundance (WT)	Abundance (KO)
PSLQTSSAR(me1)MPGSVIPPPLVRGGQQASSK	R213me1	200	0
PSLQTSSAR(me2)MPGSVIPPPLVRGGQQASSK	R213me2	200	0
MPGSVIPPPLVR(me1)GGQQASSK	R225me1	200	0
LGPQASSQVVMPPLVR(me1)	R249me1	200	0
QHSSTGPPPLLLAPR(me1)	R273me1	200	0
IIQQGLIR(me1)	R293me1	200	0

B

Peptides with arginine methylation	Type of methylation	Abundance (WT)	Abundance (KO)
SATNTTLPHMLMSQR(me1)	R264me1	200	0
VIAPNPAQLQGQR(me1)	R277me1	200	0
VIAPNPAQLQGQR(me1)GPPK	R277me1	200	0

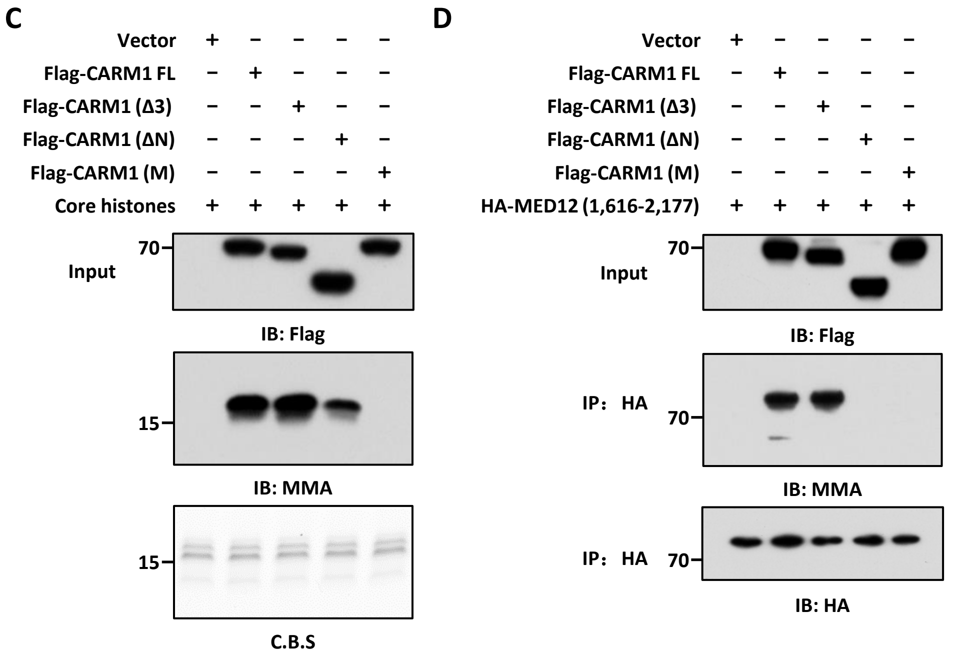

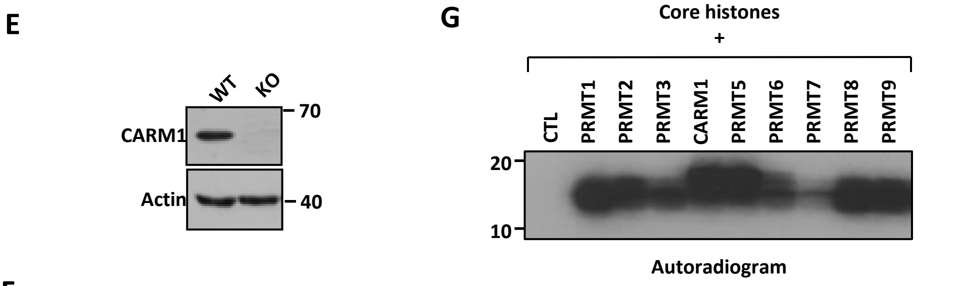

F

>NP_060130 GATAD2A

MTEEACRTRSQKRALERDPTEDDVESKKIKMERGLLASDLNTDGDMRVTPEPGAGPTQGLLRATEATA
MAMGRGEGLVGDGPVDMRTSHSDMKSERRPPSPDVIVLSDNEQPSSPRVNGLTTVALKETSTEALMKS
SPEERERMIKQLKEELRLEEAKLVLLKKLRQSQIQKEATAQKPTGSVGSTVTTPPPLVRGTQNIPAGK
PSLQTSSA**R**MPGSVIPPPLV**R**GGQQASSKLGPQASSQVVMPPLV**R**GAQQIHSI**R**QHSSTGPPPLLLAP
RASVPSVQIQGQ**R**IIQQGLI**R**VANVPNTSLLVNIPQPTPASLKGTTATSAQANSTPTSVASVVTSAES
PASRQAAAKLALRKQLEKTLLEIPPPKPPAPEMNFLPSAANNEFIYLVGLEEVVQNLLETQAGRMSAA
TVLSREPYMCAQCKTDFTCRWREEKSGAIMCENCMTTNQKKALKVEHTSRLKAAFVKALQQEQEIEQR
LLQQGTAPAQAKAEPTAAPHPVLKQASSQLSRGSATTPRGVLHTFSPSPKLQNSASATALVSRTGRHS
ERTVSAGKGSATSNWKKTPLSTGGTLAFVSPSLAVHKSSSAVDRQREYLLDMIPPRSIPQSATWK

Figure S2

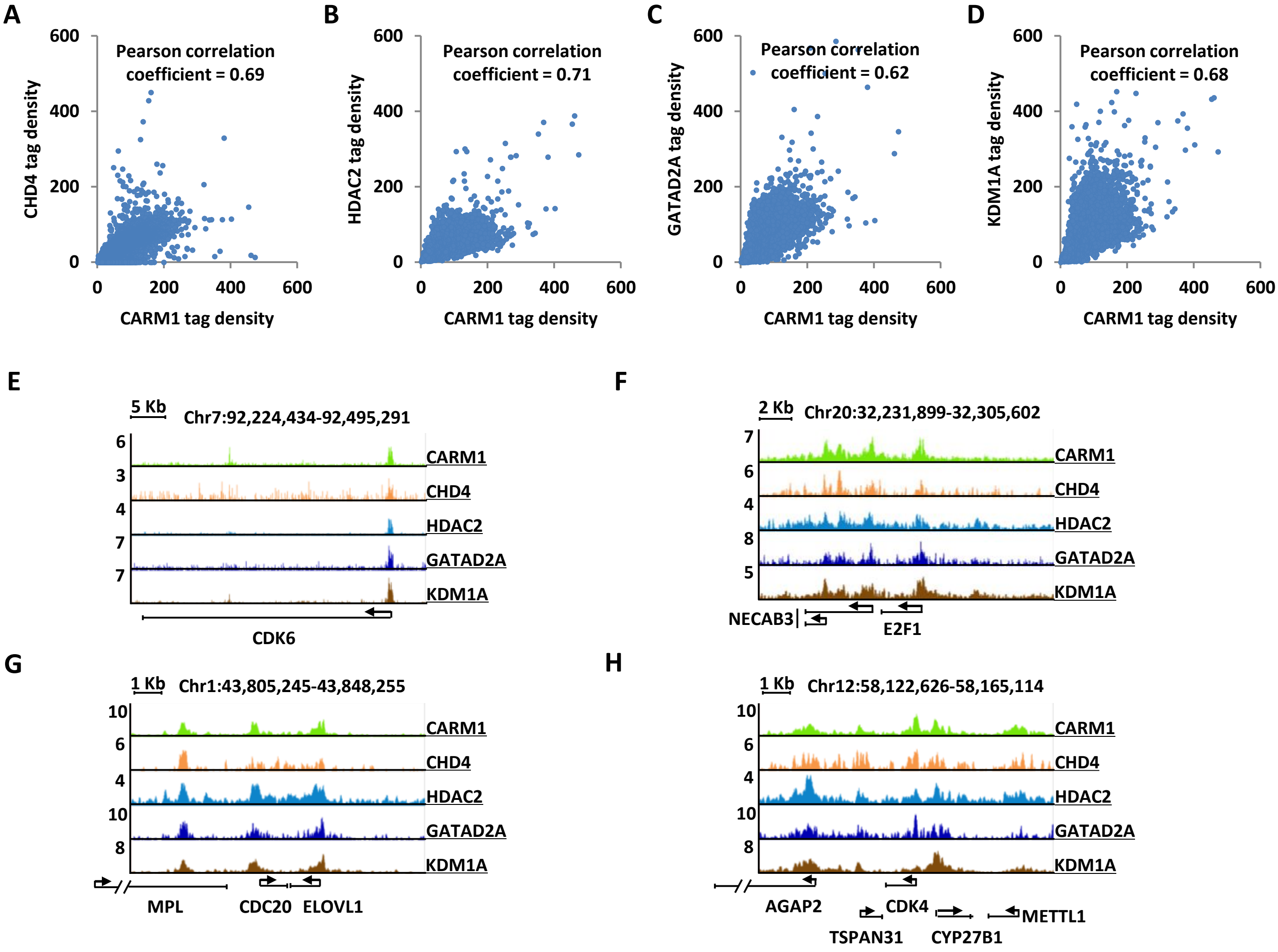

A
B
C
D
Pearson correlation coefficient = 0.69
Pearson correlation coefficient = 0.71
Pearson correlation coefficient = 0.62
Pearson correlation coefficient = 0.68
CHD4 tag density
HDAC2 tag density
GATAD2A tag density
KDM1A tag density
CARM1 tag density
E
F
G
H
5 Kb
Chr7:92,224,434-92,495,291
CDK6
2 Kb
Chr20:32,231,899-32,305,602
NECAB3
E2F1
1 Kb
Chr1:43,805,245-43,848,255
MPL
CDC20
ELOVL1
1 Kb
Chr12:58,122,626-58,165,114
AGAP2
CDK4
TSPAN31
CYP27B1
METTL1
CARM1
CHD4
HDAC2
GATAD2A
KDM1A

Figure S3

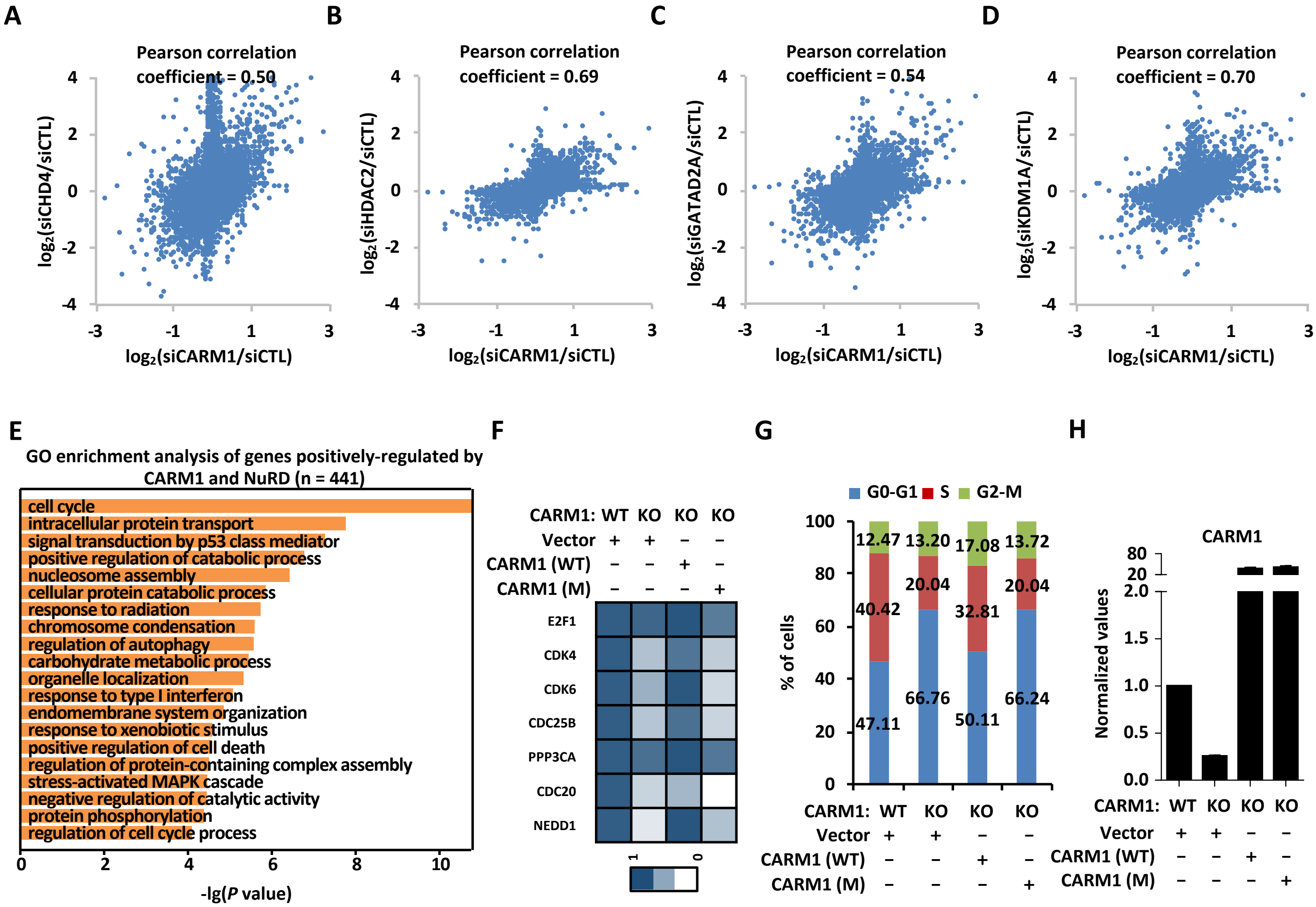

A
Pearson correlation coefficient = 0.50
log2(siCHD4/siCTL)
log2(siCARM1/siCTL)
B
Pearson correlation coefficient = 0.69
log2(siHDAC2/siCTL)
log2(siCARM1/siCTL)
C
Pearson correlation coefficient = 0.54
log2(siGATAD2A/siCTL)
log2(siCARM1/siCTL)
D
Pearson correlation coefficient = 0.70
log2(siKDM1A/siCTL)
log2(siCARM1/siCTL)
E
GO enrichment analysis of genes positively-regulated by CARM1 and NuRD (n = 441)
cell cycle
intracellular protein transport
signal transduction by p53 class mediator
positive regulation of catabolic process
nucleosome assembly
cellular protein catabolic process
response to radiation
chromosome condensation
regulation of autophagy
carbohydrate metabolic process
organelle localization
response to type I interferon
endomembrane system organization
response to xenobiotic stimulus
positive regulation of cell death
regulation of protein-containing complex assembly
stress-activated MAPK cascade
negative regulation of catalytic activity
protein phosphorylation
regulation of cell cycle process
-lg(P value)
F
CARM1: WT KO KO KO
Vector + + – –
CARM1 (WT) – – + –
CARM1 (M) – – – +
E2F1
CDK4
CDK6
CDC25B
PPP3CA
CDC20
NEDD1
1
0
G
G0-G1 S G2-M
% of cells
12.47 13.20 17.08 13.72
40.42 20.04 32.81 20.04
47.11 66.76 50.11 66.24
CARM1: WT KO KO KO
Vector + + – –
CARM1 (WT) – – + –
CARM1 (M) – – – +
H
CARM1
Normalized values
CARM1: WT KO KO KO
Vector + + – –
CARM1 (WT) – – + –
CARM1 (M) – – – +

Figure S4

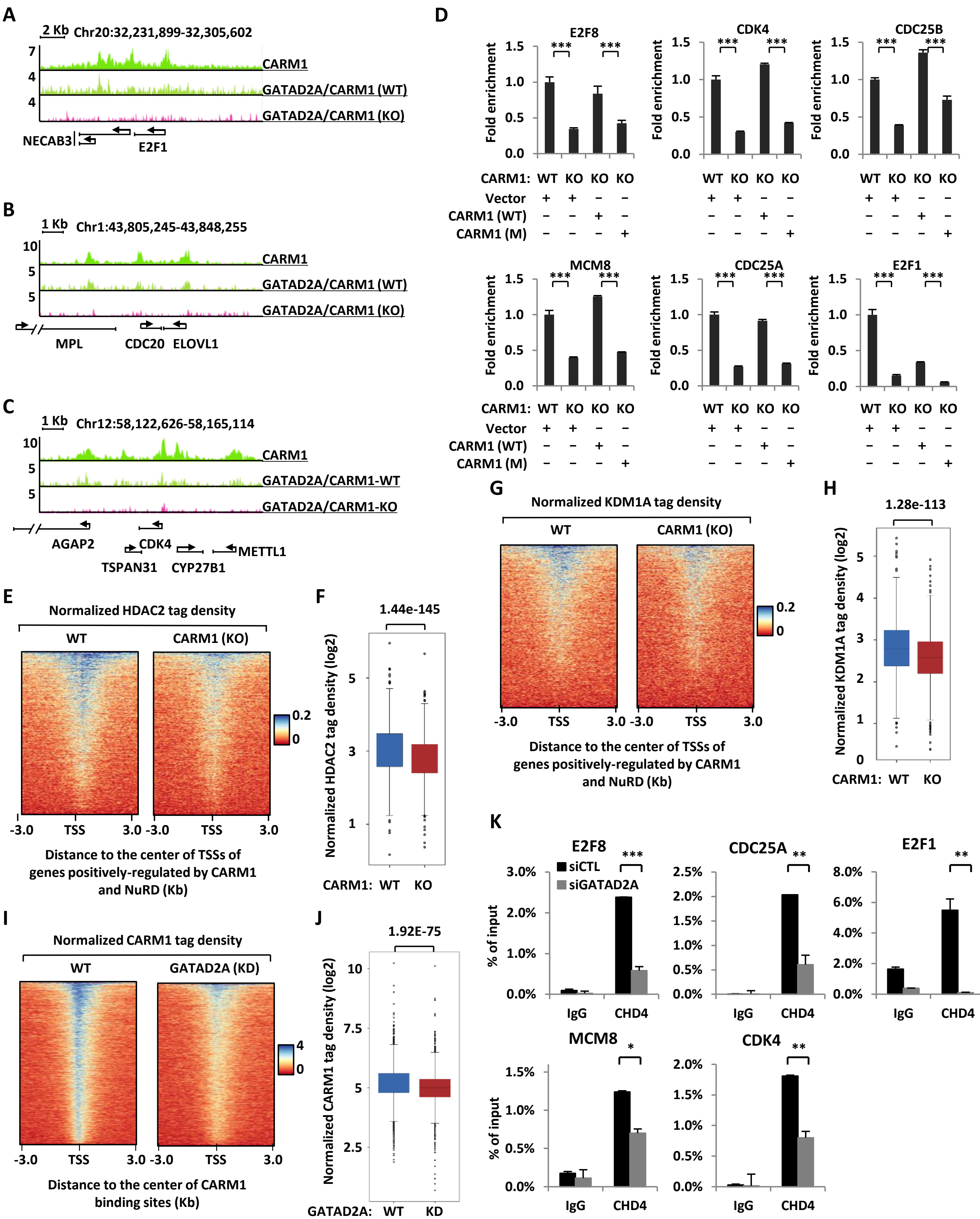
A
2 Kb Chr20:32,231,899-32,305,602
CARM1
GATAD2A/CARM1 (WT)
GATAD2A/CARM1 (KO)
NECAB3
E2F1
B
1 Kb Chr1:43,805,245-43,848,255
CARM1
GATAD2A/CARM1 (WT)
GATAD2A/CARM1 (KO)
MPL
CDC20 ELOVL1
C
1 Kb Chr12:58,122,626-58,165,114
CARM1
GATAD2A/CARM1-WT
GATAD2A/CARM1-KO
AGAP2
CDK4
TSPAN31
CYP27B1
METTL1
D
E2F8
CDK4
CDC25B
MCM8
CDC25A
E2F1
Fold enrichment
CARM1: WT KO KO KO
Vector
CARM1 (WT)
CARM1 (M)
E
Normalized HDAC2 tag density
WT
CARM1 (KO)
Distance to the center of TSSs of genes positively-regulated by CARM1 and NuRD (Kb)
F
1.44e-145
Normalized HDAC2 tag density (log2)
CARM1: WT KO
G
Normalized KDM1A tag density
WT
CARM1 (KO)
Distance to the center of TSSs of genes positively-regulated by CARM1 and NuRD (Kb)
H
1.28e-113
Normalized KDM1A tag density (log2)
CARM1: WT KO
I
Normalized CARM1 tag density
WT
GATAD2A (KD)
Distance to the center of CARM1 binding sites (Kb)
J
1.92E-75
Normalized CARM1 tag density (log2)
GATAD2A: WT KD
K
E2F8
CDC25A
E2F1
MCM8
CDK4
siCTL
siGATAD2A
% of input
IgG
CHD4

Figure S5

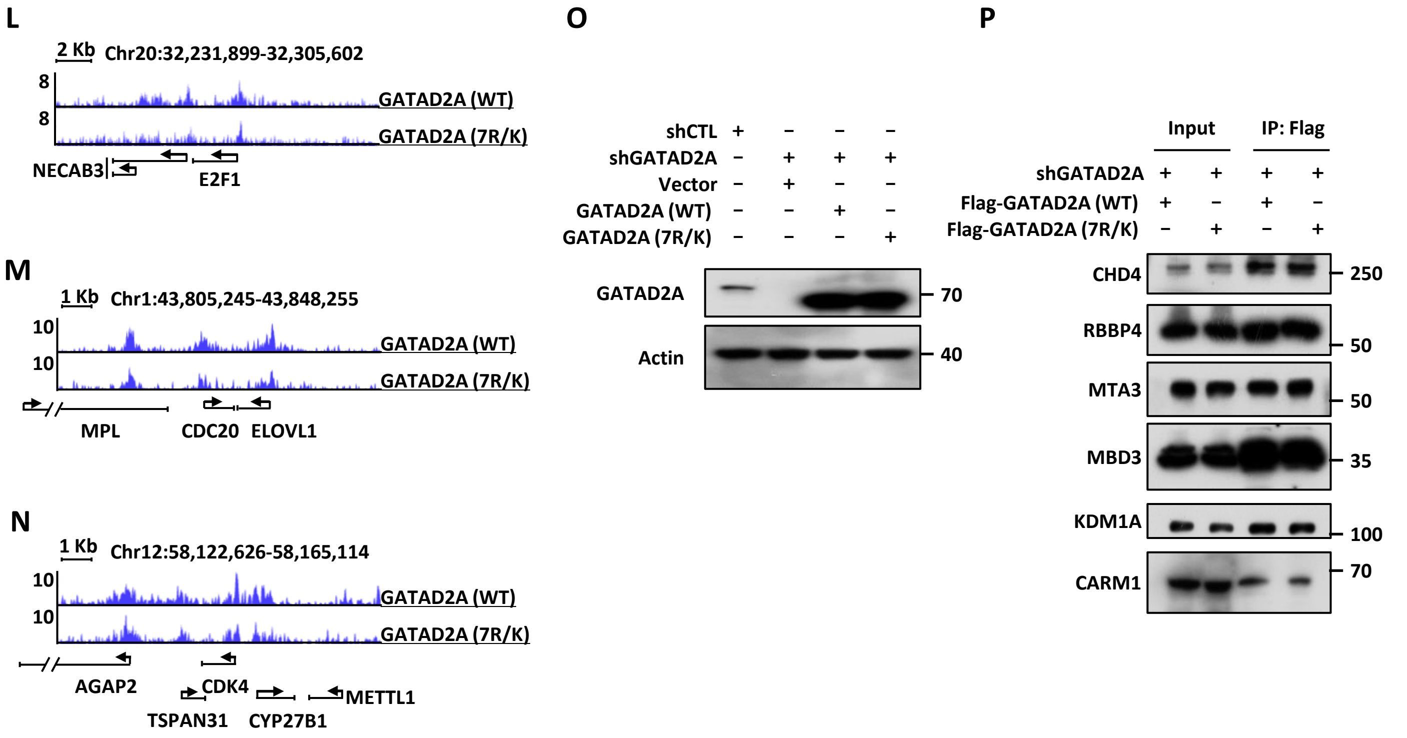

Figure S5 cont'

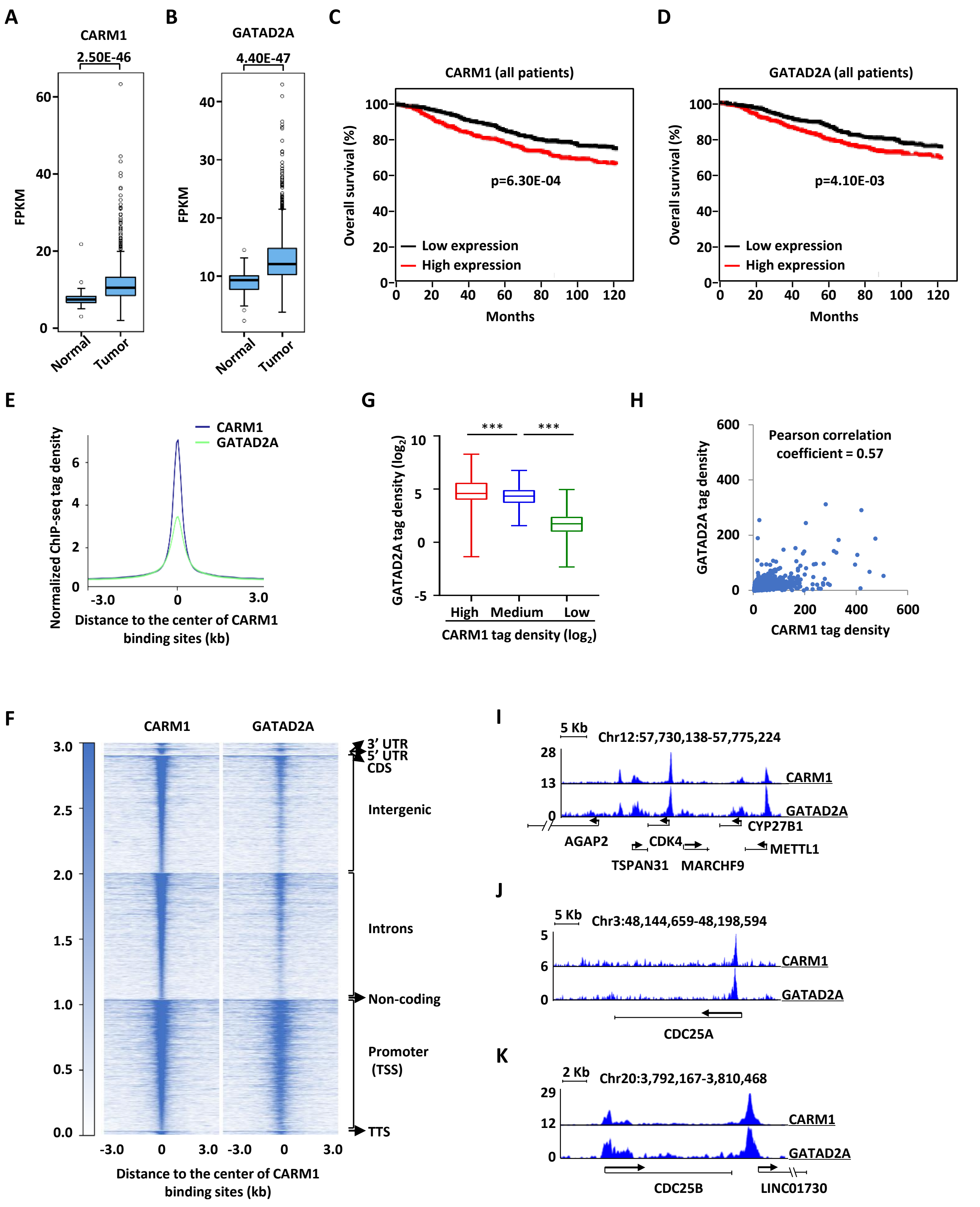
A
CARM1
2.50E-46
FPKM
Normal
Tumor
B
GATAD2A
4.40E-47
C
CARM1 (all patients)
Overall survival (%)
p=6.30E-04
Low expression
High expression
Months
D
GATAD2A (all patients)
p=4.10E-03
E
CARM1
GATAD2A
Normalized ChIP-seq tag density
Distance to the center of CARM1 binding sites (kb)
G
GATAD2A tag density (log2)
High
Medium
Low
CARM1 tag density (log2)
H
Pearson correlation coefficient = 0.57
GATAD2A tag density
CARM1 tag density
F
3' UTR
5' UTR
CDS
Intergenic
Introns
Non-coding
Promoter (TSS)
TTS
I
5 Kb
Chr12:57,730,138-57,775,224
AGAP2
CDK4
CYP27B1
TSPAN31
MARCHF9
METTL1
J
Chr3:48,144,659-48,198,594
CDC25A
K
2 Kb
Chr20:3,792,167-3,810,468
CDC25B
LINC01730

Figure S6

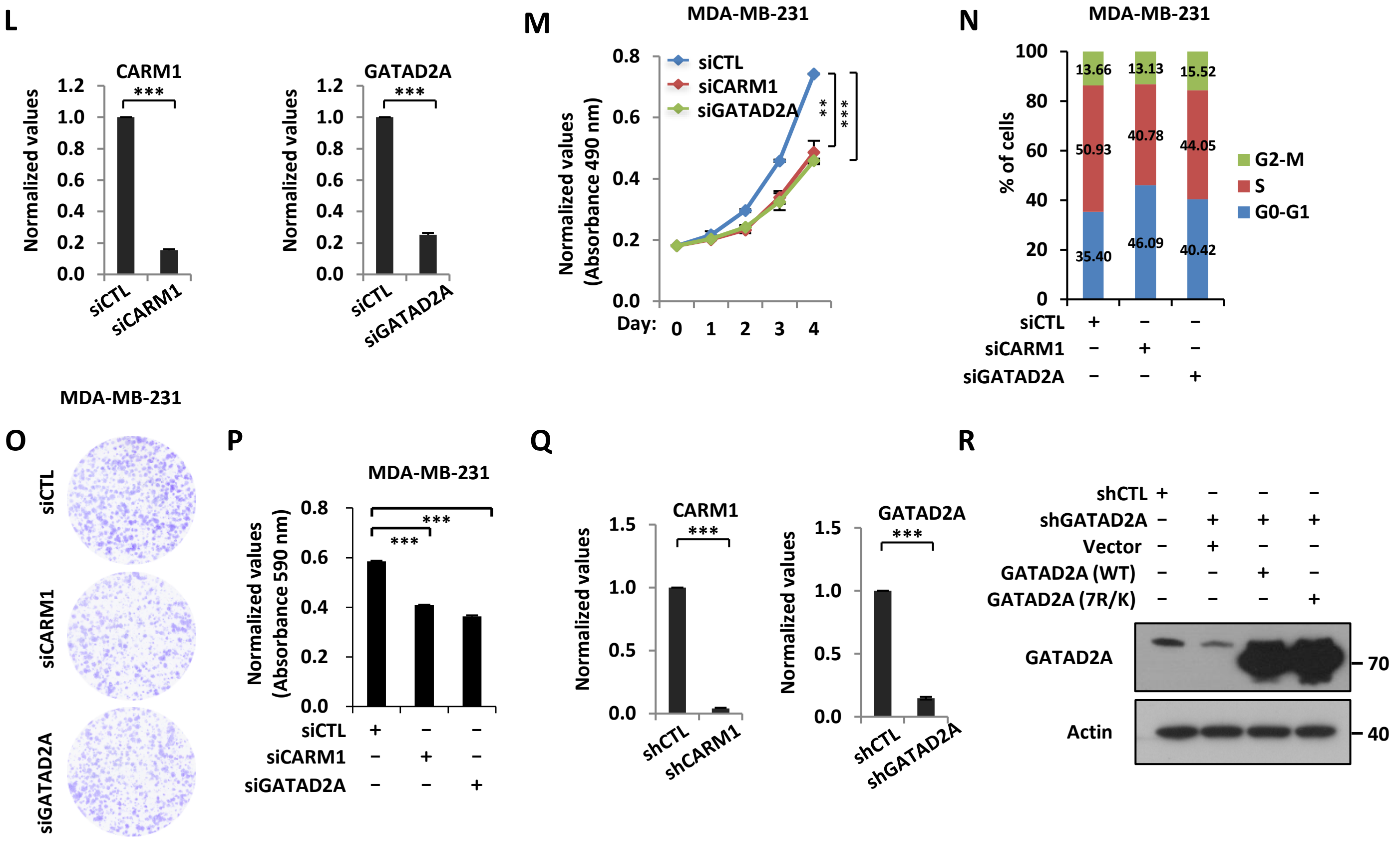

Figure S6 Cont'd

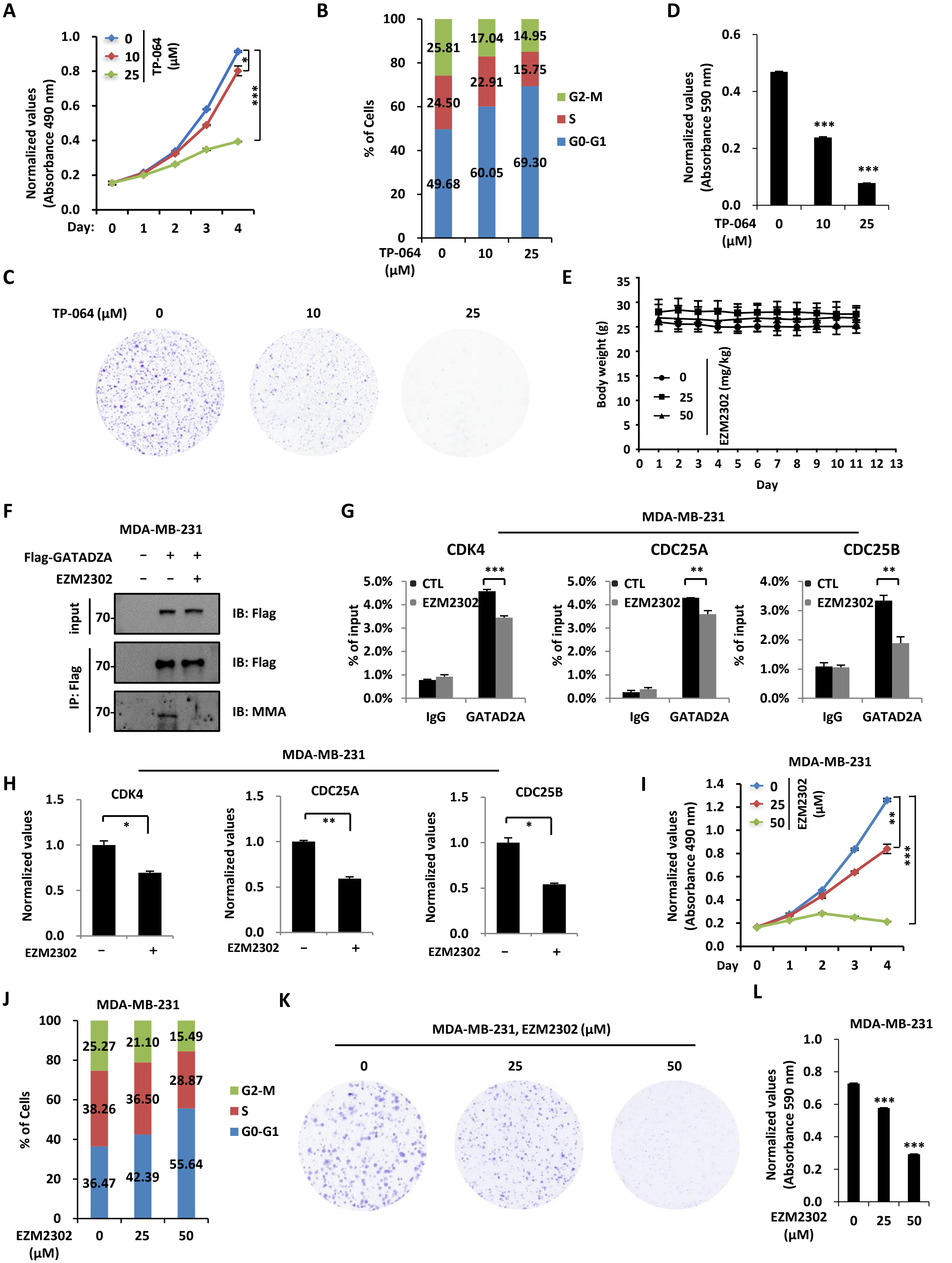
A
B
C
D
E
F
G
H
I
J
K
L
TP-064 (μM)
Normalized values (Absorbance 490 nm)
Day:
% of Cells
G2-M
S
G0-G1
Normalized values (Absorbance 590 nm)
Body weight (g)
EZM2302 (mg/kg)
Day
MDA-MB-231
Flag-GATADZA
EZM2302
input
IP: Flag
IB: Flag
IB: MMA
CDK4
CDC25A
CDC25B
CTL
% of input
IgG
GATAD2A
Normalized values
MDA-MB-231, EZM2302 (μM)
EZM2302 (μM)

Figure S7

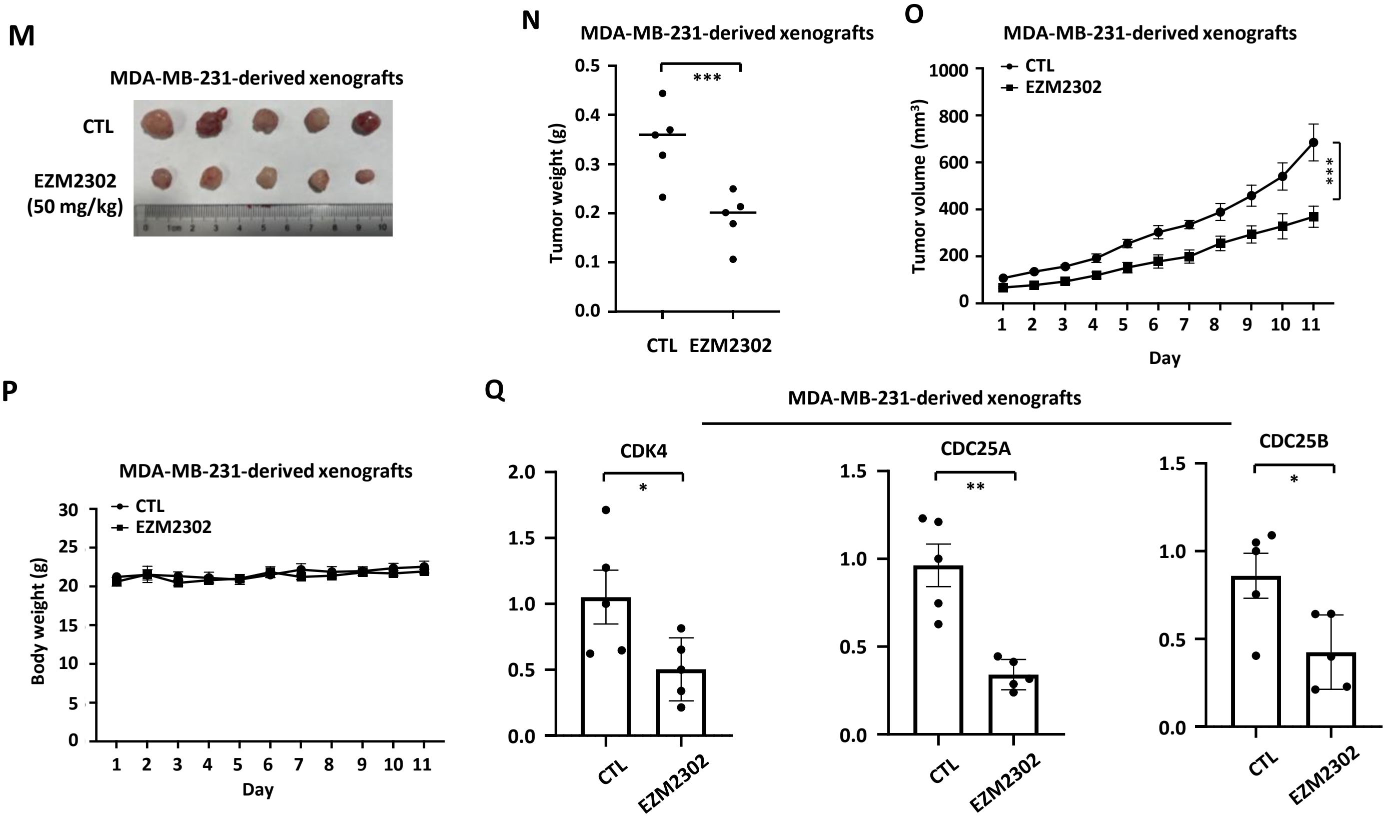

Figure S7 Cont'd
